# A novel approach for relapsed/refractory FLT3^mut+^ acute myeloid leukaemia: synergistic effect of the combination of bispecific FLT3scFv/NKG2D-CAR T cells and gilteritinib

**DOI:** 10.1186/s12943-022-01541-9

**Published:** 2022-03-04

**Authors:** Ke-xin Li, Hui-yang Wu, Wan-ying Pan, Meng-qi Guo, De-zhi Qiu, Yan-jie He, Yu-hua Li, Dong-Hua Yang, Yu-xian Huang

**Affiliations:** 1 Department of Hematology, Zhujiang Hospital, Southern Medical University, Guangzhou, 510282 Guangdong China; 2 Department of Pharmaceutical Sciences, College of Pharmacy and Health Sciences, St. John’s University, Queens, NY 11439 USA

**Keywords:** Acute myeloid leukaemia, Chimeric antigen receptor (CAR), Immunotherapy, Natural killer group 2 member D (NKG2D), FMS-like tyrosine kinase 3 (FLT3), FLT3 inhibitor, Gilteritinib

## Abstract

**Background:**

Patients with relapsed/refractory acute myeloid leukaemia (AML) with FMS-like tyrosine kinase 3-internal tandem duplication (FLT3-ITD) have limited treatment options and poor prognosis. Therefore, novel treatment modalities are needed. Since high expression of natural killer group 2 member D ligands (NKG2DLs) can be induced by FLT3 inhibitors, we constructed dual-target FLT3 single-chain fragment variable (scFv)/NKG2D-chimeric antigen receptor (CAR) T cells, and explored whether FLT3 inhibitors combined with FLT3scFv/NKG2D-CAR T cells could have synergistic anti-leukaemia effects.

**Methods:**

FLT3scFv and NKG2D expression in CAR T cells, FLT3 and NKG2DL expression in AML cells, and the in vitro cytotoxicity of combining CAR T cells with gilteritinib were assessed by flow cytometry. The therapeutic effect was evaluated in a xenograft mouse model established by injection of MOLM-13 cells. Mechanisms underlying the gilteritinib-induced NKG2DL upregulation were investigated using siRNA, ChIP-QPCR and luciferase assays.

**Results:**

The FLT3scFv/NKG2D-CAR T cells specifically lysed AML cells both in vitro and in the xenograft mouse model. The efficacy of FLT3scFv/NKG2D-CAR T cells was improved by gilteritinib-pretreatment. The noncanonical NF-κB2/Rel B signalling pathway was found to mediate gilteritinib-induced NKG2DL upregulation in AML cells.

**Conclusions:**

Bispecific FLT3scFv/NKG2D-CAR T cells can effectively eradicate AML cells. The FLT3 inhibitor gilteritinib can synergistically improve this effect by upregulating NF-κB2-dependent NKG2DL expression in AML cells.

**Graphical Abstract:**

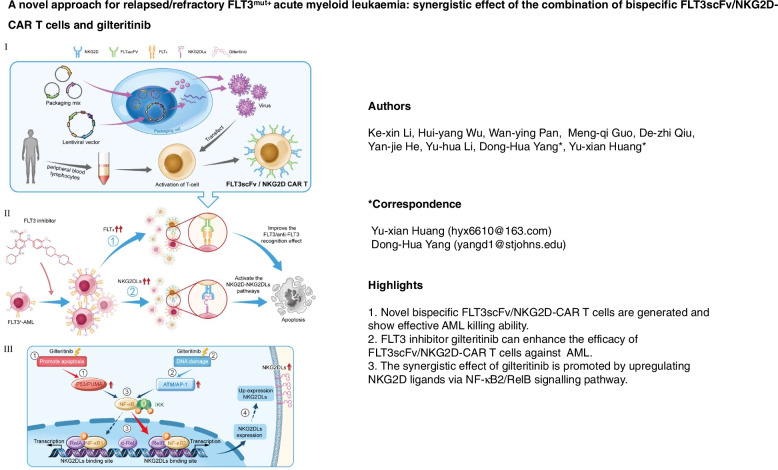

**Supplementary information:**

The online version contains supplementary material available at 10.1186/s12943-022-01541-9.

## Background

FMS-like tyrosine kinase 3 (FLT3), also known as stem cell tyrosine kinase-1 (STK-1), belongs to the type III receptor tyrosine kinase family. FLT3 is a proto-oncogene located on chromosome band 13q12 [1]. FLT3 mutations are present in approximately 30% of patients with de novo acute myeloid leukaemia (AML) [2]. Of these mutations, 20–25% are FLT3 internal tandem duplication (FLT3-ITD) mutations, and the other 5–10% are FLT3 tyrosine kinase domain (FLT3-TKD) mutations [3]. ITD mutations interfere with the negative regulatory function of the juxtamembrane region, and kinase domain point mutations involve the activation loop. These mutations result in loss of autoinhibitory function with subsequent constitutive activation of FLT3 kinase and its downstream proliferative signalling cascades involving JAK/STAT, MAPK, RAS, MEK, AKT/ERK, and PI3K, leading to sustainable proliferation and differentiation of leukemic cells [4]. These factors contribute to a poor prognosis and high risk of relapse of AML following induction/consolidation chemotherapy and allogeneic haematopoietic stem cell transplantation (HSCT) [5–8]. FLT3-ITD mutations are particularly associated with a high recurrence rate and low 5-year overall survival in patients with relapsed/refractory AML, with a five-year event-free survival (EFS) of 12% and an overall survival (OS) of 16.6% [9, 10]. Thus, treatment of relapsed/refractory FLT3^mut+^ AML remains one of the greatest challenges in AML management, requiring innovative treatment strategies.

The high frequency of FLT3^mut+^ in relapsed/refractory AML suggests that FLT3 is an ideal target for patients with relapsed/refractory AML. This idea has led to the development of a variety of FLT3 inhibitors, including the first-generation nonselective inhibitors such as sunitinib, sorafenib and midostaurin and the second-generation selective inhibitors, including crenolanib, gilteritinib, and quizartinib [4]. To date, the FDA has approved midostaurin and gilteritinib for patients with FLT3^mut+^ AML, but only gilteritinib has been approved as a monotherapy for relapsed/refractory FLT3^mut+^ AML based on the beneficial outcomes of ADMIRAL clinical trials [11, 12]. However, the effectiveness of gilteritinib is limited, with a low remission rate and short sustained remission time, although it significantly prolongs the OS compared with chemotherapy [4, 11].

Following the success of CD19-chimeric antigen receptor (CAR) T cell therapy for relapsed or refractory large B cell lymphoma, much effort has been made to translate this therapeutic approach into AML management [13–15]. CAR T cells targeting CD123, CLL1, et al. have been developed and have entered into phase I/II clinical trials for AML patients [15]. FLT3-CAR T cells have been developed in the preclinical stage, and data from studies using FLT3^mut+^ AML xenograft mice have demonstrated that FLT3-CAR T cells can significantly prolong the survival of the mice [16, 17]. However, AML target antigen escape, lack of specific antigen and CAR-T cell therapy related toxicity after CAR T cell therapy are the main obstacles restricting the treatment of refractory AML with CAR-T cells [18]. One of the approaches for overcoming this problem is to target more than one antigen on cancer cells simultaneously.

The NKG2D − NKG2DL signalling pathway is an important activating signal between immune effector cells and tumour cells. The expression level of NKG2DLs on tumour cells directly affects the antitumour activity of immune effector cells, such as natural killer (NK) cells, dendritic cells (DCs), cytotoxic T lymphocytes (CTLs), and others [19]. In AML cells, the expression of the NKG2DLs, including MICA/B, ULBP1/2, and ULBP3 were found to be 0–75%, 16–63%, and 16–100%, respectively. In contrast, their expression levels on normal cells were found to be extremely low, making NKG2DLs ideal targets for AML [20]. However, immune escape of leukaemia stem cells (LSCs) is one of the fundamental causes of refractory/relapsed AML. A recent study reported that a lack of NKG2DL expression on the surface of LSCs is the key factor leading to immune escape of AML [21]. Therefore, activating the NKG2D-NKG2DL signalling pathway by increasing NKG2D expression on immune cells and NKG2DL expression on AML cells can not only improve the tumour-killing ability of CAR T cells but also prevent AML relapse. We previously found that the FLT3 inhibitors sorafenib and sunitinib blocked the signalling pathways of tumour cell proliferation and induced apoptosis of nasopharyngeal carcinoma cells [22]. In addition, these compounds increased the expression of NKG2DLs in carcinoma and hepatoma cells and eventually enhanced the killing capacity of effector T cells by activating NK, NKT and TCRαβ T cells that expressed NKG2D receptors. The combined administration of FLT3 inhibitors and NK cells achieved synergistic killing in nasopharyngeal carcinoma and hepatocellular carcinoma [22, 23]. Jetani et al. demonstrated a superior synergistic anti-leukaemia efficacy using FLT3-CAR T cells and the FLT3 inhibitor crenolanib [16]. However, whether dual-target CAR T cells targeting both FLT3 and NKG2D are effective and whether the combination of an FLT3 inhibitor and these bispecific FLT3 single-chain fragment variable (scFv)/NKG2D-CAR T cells can induce a synergistic anti-AML effect have not been assessed.

We therefore engineered primary T cells from healthy donors to generate bispecific FLT3scFv/NKG2D-CAR T cells. In vitro studies showed that FLT3scFv/NKG2D-CAR T cells had significant cytotoxicity against AML cells (MOLM-13 cells, MV4-11 cells, and bone marrow mononuclear cells from AML patients). The treatment induced substantial IL-2 and interferon gamma (IFNγ) expression. Such effects were also induced in AML-bearing mice established by MOLM-13 cell injection. CAR T cell treatment significantly prolonged the survival of mice with AML compared to control treatment. Combination with gilteritinib enhanced the AML-killing ability of FLT3scFv/NKG2D-CAR T cells, likely via the upregulation of NKG2DLs and FLT3 both in vitro and in vivo. Finally, we found that the noncanonical NF-κB2/RelB signalling pathway mediated gilteritinib-induced NKG2DL upregulation in AML cells.

## Materials and methods

### CAR design and the generation of CAR T cells

**Fig. 1:**
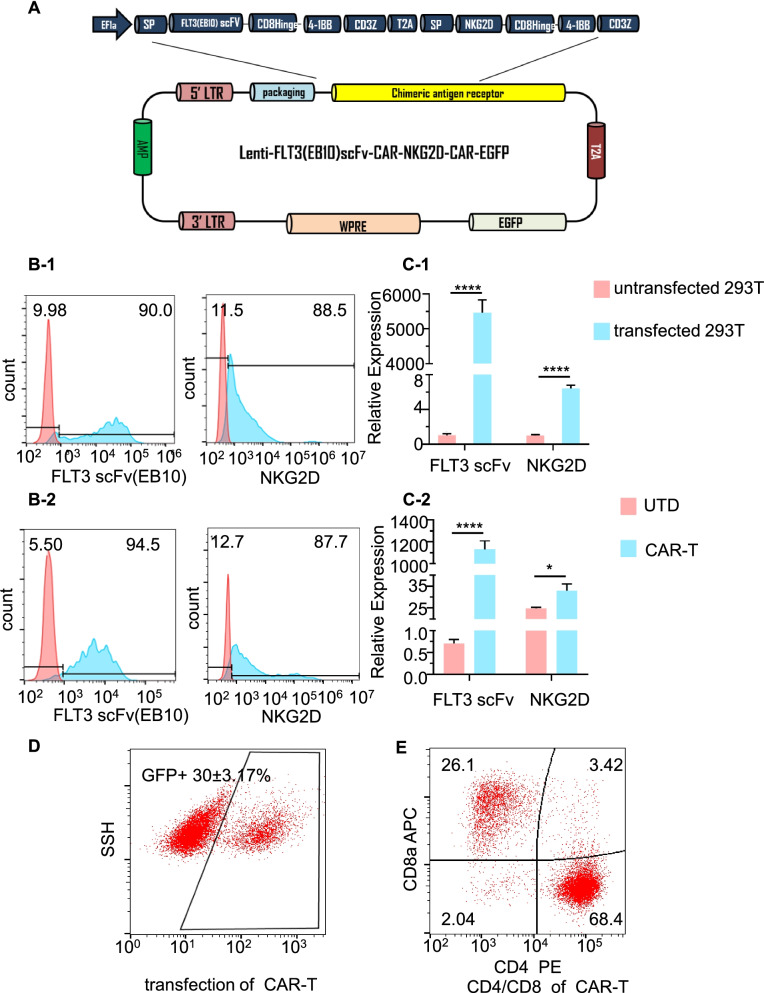
Generation of CAR T cells targeting FLT3 and NKG2DLs**. A** A schematic representation of the manufactured FLT3scFv/NKG2D-CAR lentiviral construct. SP, signal peptide; scFv, single-chain fragment variable; Hinge, hinge chain. **B** FLT3scFv (EB10) and NKG2D expression assessed by flow cytometry of GFP + -transfected 293 T cells (B-1) and GFP + CAR T cells (B-2). The histograms show staining with recombinant FLT3 protein + anti-His APC antibody or anti-human NKG2D antibody (blue) compared to staining with isotype (red). **C** FLT3scFv (EB10) and NKG2D expression assessed by real-time PCR in transfected 293 T cells (C-1) and CAR T cells (C-2) compared with untransfected 293 T cells and UTD cells, respectively. **D** Significant CAR T cell transfection efficiency indicated by the percentage of GFP^+^ cells determined by flow cytometry. **E** CD4 and CD8 subtypes of CAR T cells assessed by flow cytometry. The data shown are derived from three independent experiments with T cells from different donors. UTD, un-transduced CD3 + T cells; GFP, green fluorescent protein; SSH, side scatter height; APC, allophycocyanin; PE, phycoerythrin. * *p* < 0.05, **** *p* < 0.0001

The construction of the FLT3scFv/NKG2D-CAR lentiviral vector is shown in Fig. 1A. In brief, a second-generation dual FLT3 scFv/NKG2D-CAR was generated through a bicistronic vector, which contained two CAR units linked by the "self-cleaving" 2A peptide. Each CAR unit was composed of an extracellular domain derived from a single-chain variable fragment (scFv) of a monoclonal antibody against FLT3 or NKG2D linked to the CD3ζ chain of the TCR complex using a CD8 spacer and transmembrane domain, together with the 4-1BB costimulatory domain. The bicistronic vector was linked to the transduction marker EGFP by the T2A peptide, which was encoded in the lentivirus vector pCDH and expressed under the control of the EF1α hybrid promoter. The sequences of the extracellular domains of NKG2D, the CD8 hinge and transmembrane regions, and the CD3ζ cytoplasmic region were taken from NCBI GenBank. The sequence of FLT3scFv was based on the previously published antibody EB10 [24]. All sequences were confirmed by gene sequencing (Life Technology, China) under the control of the empty vector pCDH. The details of the sequences of NKG2D and FLT3scFv are shown in Supplementary Table 1 and the generation of CAR T cells are shown in Supplementary methods

### Cell lines and culture

Bone marrow samples were obtained from AML patients (5 patients with FLT3^mut+^ AML and 5 patients with FLT3 ^mut−^ AML) hospitalized in Zhujiang Hospital of Southern Medical University. Neonatal cord blood (*n* = 3) was obtained from the Guangdong Cord Blood Bank. The Ethics Committee of Zhujiang Hospital of Southern Medical University approved the study, which was in accordance with the guidelines of the Declaration of Helsinki. The characteristics of patients are summarized in Supplementary Table 2. Peripheral blood and bone marrow mononuclear cells were isolated using Ficoll-Paque density gradient medium (TBD Science, China) and kept in liquid nitrogen for CAR T cell construction and cellular studies.

AML cell lines (MOLM-13 and MV4-11) and 293T cells were provided by the Haematology Laboratory at Zhujiang Hospital of Southern Medical University. 293T cells were cultured in high-glucose DMEM containing 10% FBS (Millipore, USA), 100 U/ml penicillin, and 100 mg/ml streptomycin (Life Technologies, USA). MOLM-13 cells were cultured in 1640 medium containing 10% FBS (Gibco, USA), 100 U/ml penicillin, and 100 mg/ml streptomycin (Life Technologies, USA). MV4-11 cells were cultured in IMDM containing 15% FBS. All AML cells were cultured in a humidified incubator with 5% CO_2_ at 37 °C.

Some MOLM-13 and MV4-11 cells were pre-treated with gilteritinib (Selleck Chemical, USA) at the IC25 concentration for 24 h. The IC25 concentration produced a clinically achievable serum level and was used in the subsequent experiments unless otherwise specified.

### Flow cytometry

Flow cytometry was used to detect the expression of NKG2DLs and FLT3 on AML cells, CAR expression, CAR T cell subsets, the effect of gilteritinib on CAR T cells and CAR T cell cytotoxicity against AML cells. Cells were harvested, washed twice with 1 × PBS, and resuspended in PBS (at a density of 1 × 10^6^ cells/ml). Subsequently, primary antibodies were added to the cell suspension according to the manufacturer’s instructions and incubated for 30 min at 4 °C in the dark. After incubation, the cells were washed three times with ice-cold PBS, and flow cytometry was performed using a Canto II flow cytometer (BD Biosciences, USA). To assess FLT3scFv expression, 1600 nM FLT3-His (Sino Biological, China) recombinant protein was added to the cells (1 × 10^5^ cells) and incubated on ice for 1 h; 1 ml PBS was added, and the samples were centrifuged at 3500 rpm for 5 min. After the supernatant was discarded, 2 µl of anti-His tag APC secondary antibody (Biolegend, USA) was added. The sample was incubated at 4 °C for 30 min, and then the cells were washed with PBS. GFP + cells expressing FLT3scFv were detected with the Canto II flow cytometer (BD Biosciences, USA). The percentage of CD33 + cells (MOLM13, MV4-11 and primary AML cells) was representative of the survival level of target cells [17]. The data were analysed using FlowJo 7.6.5 software (Tree star Inc. Ashland, OR, USA). All the antibodies used are listed in Supplementary Table 3.

### Xenograft mouse model

Animal experiments were performed using protocols approved by the Ethics Committee of Zhujiang Hospital of Southern Medical University. All experiments followed the regulatory standards. Female 6- to 8-week-old NOD/SCID IL-2RγCnull (NSG) mice were purchased from Biocytogen. Mice were bred under specific pathogen-free conditions and randomized into 4 groups: (1) control, (2) gilteritinib, (3) FLT3scFv/NKG2D-CAR T cell and (4) combination of FLT3scFv/NKG2D-CAR T cell and gilteritinib. On day zero, luciferase-transfected MOLM-13 cells (2 × 10^6^ cells/ml) were injected via the tail vein of all mice. Starting on day 7, gilteritinib was dissolved in 4% DMSO and then administered by intraperitoneal injection at a dose of 15 mg/kg 5 days per week for 3 weeks in Groups 2 and 4. From day 8, a single dose of FLT3scFv/NKG2D-CAR T cells (2.5 × 10^6^ cells) based on the published reported [25] was injected via the tail vein in Groups 3 and 4. Leukaemia progression was monitored by serial bioluminescence (BL) imaging using an IVIS Lumina imaging system (PerkinElmer, USA) following D-luciferin substrate administration (0.3 mg/g body weight, i.p.) (Promega, USA). Data were analysed using Bruker MI SE (Bruker FX Pro, USA). Survival curves were generated.

### Statistical analysis

Experiments were repeated at least three times unless specified otherwise in the text. Data are expressed as the mean ± S.E.M. and were analysed using SPSS 24.0 software (SPSS Inc., Chicago, IL, USA). Student’s t-test was used for comparisons between two groups, and one-way ANOVA was used for comparisons among multiple groups. Kaplan–Meier survival analysis was performed to assess survival differences among the treatment groups, and the p value was calculated using the log-rank test. *p* < 0.05 was defined as statistically significant.

## Results

### *Bispecific FLT3scFv/NKG2D-CAR T cells eliminate AML cells *in vitro

We developed dual CAR T cells specifically targeting NKG2DLs and FLT3 on the AML cell membrane (Fig. 1A). To achieve this, vectors encoding NKG2D-CAR and FLT3scFv-CAR were first constructed and then transduced into 293T cells to produce lentiviruses simultaneously expressing NKG2D and FLT3scFv. Finally, the viruses were transduced into T cells isolated from three healthy donors to develop FLT3scFv/NKG2D-CAR T cells. To confirm the successful engineering of CAR T cells, NKG2D and FLT3scFv expression on 293T cells and engineered T cells was evaluated using flow cytometry and Q-PCR analysis. The results showed substantial expression of both NKG2D and FLT3scFv on the cell surface of transfected 293T cells and CAR T cells (all *p* < 0.05 compared with nontransduced 293T cells and CD3 + T cells) (Fig. 1B and C, Supplementary Fig. 8 A-B). GFP-positive CAR T cells accounted for 30 ± 3.17% (Fig. 1D). The CAR T cells consisted of both CD4 + and CD8 + T cells. The CD4 + /CD8 + ratio was 2.6 (68.4 ± 2.1/26.1 ± 3.4) (Fig. 1E, Supplementary Fig. 8C).

**Fig. 2:**
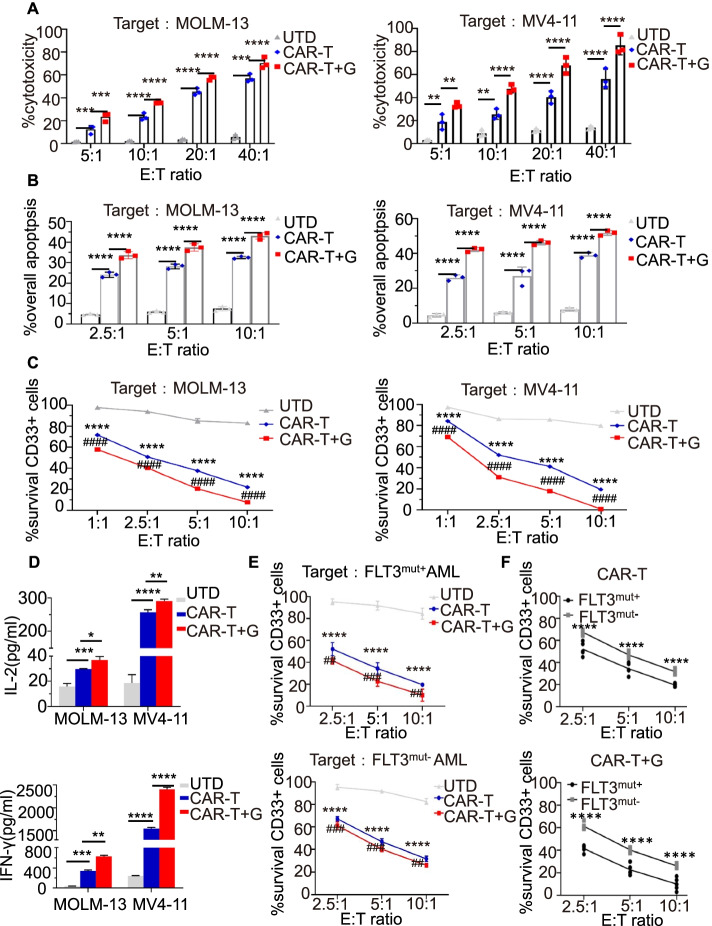
Gilteritinib enhanced the ability of FLT3scFv/NKG2D-CAR T cells in killing AML cells in vitro.** A** Special cytotoxicity of UTD, FLT3scFv/NKG2D CAR T alone or combined with gilteritinib against AML cells (MOLM-13 and MV4-11) using LDH cytotoxicity assay (4-h co-culture with 10,000 target cells/well). **B** Annexin V/PI apoptosis assay (48-h co-culture with 2 × 10^5^ target cells/well) showing FLT3scFv/NKG2D-CAR T cells had potent and specific cytotoxicity and apoptotic effects against MOLM-13 and MV4-11 cells. These effects were enhanced by pretreatment with the FLT3 inhibitor gilteritinib. **C** Flow cytometry analysis showing the percentage of surviving CD33 + AML cells (24-h co-culture of MOLM-13 and MV4-11 with 25,000 cells/well) significantly reduced in the FLT3scFv/NKG2D-CAR T cell group compared with the UTD group in a dose-dependent manner, and the gilteritinib combination treatment group showed a further decrease in the percentage of residual CD33 + AML cells. **D** ELISA analyses showing IL-2 and IFN-γ secretion by UTD and FLT3scFv/NKG2D-CAR T cells after 24-h incubation with non-treated or pre-treated target cells (MOLM-13 and MV4-11) at an E:T ratio of 5:1 (T cells: 2.5 × 10^6^/well; target cells: 5 × 10^5^/well).The levels of secreted IL-2 and IFN-γ were further increased by gilteritinib pre-treatment for 24 h. **E** Flow cytometry analysis showed the survival of AML cells from patients with FLT3^mut+^ or FLT3^mut−^ AML (24-h co-culture with 25,000 cells/well) reduced with FLT3scFv/NKG2D-CAR T cell treatment in a dose-dependent manner and further decreased in AML cells pre-treated with gilteritinib. **F** The diagrams show fewer surviving CD33 + cells in FLT3^mut+^ AML cells than in FLT3^mut−^ cells following FLT3scFv/NKG2D-CAR T cell therapy with or without gilteritinib pre-treatment. G, gilteritinib; LDH, lactate dehydrogenase; UTD: un-transduced CD3 + T cells. * *p* < 0.05; ** and ## *p* < 0.01; *** and ### *p* < 0.001; **** and #### *p* < 0.0001

To assess the killing capacity of CAR T cells in vitro, we cocultured FLT3scFv/NKG2D-CAR T cells or untransduced (UTD) T cells with two FLT3 ^mut+^AML cell lines (MOLM-13 and MV4-11). FLT3scFv/NKG2D CAR T cells showed more binding ability to FLT3 ^mut+^AML cells (Supplementary Fig. 7) and displayed greater proliferation potential after 5 days of culture compared with untransduced (UTD) T cells (Supplementary Fig. 6B). A substantial increase in cytotoxicity and apoptosis and a significant decrease in the survival rate of CD33^+^-marked MOLM-13 and MV4-11 cells were found following CAR T cell treatment in a dose-dependent manner (all *p* < 0.01) (Fig. 2A-C). FLT3scFv/NKG2D CAR T cells also showed certain cytotoxicity against different FLT3^mut−^ AML cell lines (K562 and U937 cells) (Supplementary Fig. 4), while FLT3scFv/NKG2D CAR T cells revealed more killing activity against FLT3 ^mut+^AML cell lines. We also found that gilteritinib could significantly increase the secretion of IL-2 and IFN-γ by FLT3scFv/NKG2D-CAR T cells stimulated by AML cell lines (MOLM-13 and MV4-11) (Fig. 2D). The same antitumour effects of the CAR T cells were also detected with primary AML cells, and there was a significantly reduced survival rate of bone marrow mononuclear cells taken from patients with FLT3^mut+^ or FLT3^mut−^ AML (Fig. 2E). Although the CAR T cells targeted both types of AML cells, the survival rate of FLT3 ^mut+^ AML cells was lower than that of FLT3 ^mut−^ AML cells (*p* < 0.0001) (Fig. 2F), indicating that FLT3scFv/NKG2D-CAR T cells eradicated more FLT3 ^mut+^ AML cells than FLT3 ^mut−^ AML cells.

### Gilteritinib increases the therapeutic activity of FLT3scFv/NKG2D-CAR T cells against AML cells

Gilteritinib was approved by the FDA as monotherapy for adult patients with relapsed or refractory FLT3-ITD AML [12]. The gilteritinib-mediated cytotoxicity was shown in Supplementary Fig. 3. We hypothesized that combined administration of CAR T cells with gilteritinib could enhance tumour killing via a multimodal killing mechanism. To test this hypothesis, AML cells were pretreated with gilteritinib prior to CAR T cell treatment. We found that the combination outperformed the killing effect of CAR T cell monotherapy, as indicated by the significantly increased cytotoxicity and apoptosis and decreased survival rate of CD33 + AML cells (all *p* < 0.01) (Fig. 2A-C, E, F). The expression levels of IL-2 and IFN-γ were further increased with the combined treatment compared to CAR T cell monotherapy (all *p* < 0.01) (Fig. 2D). These data demonstrate that gilteritinib synergistically enhances the antitumour effects of FLT3scFv/NKG2D-CAR T cells against AML cells in vitro.

**Fig. 3:**
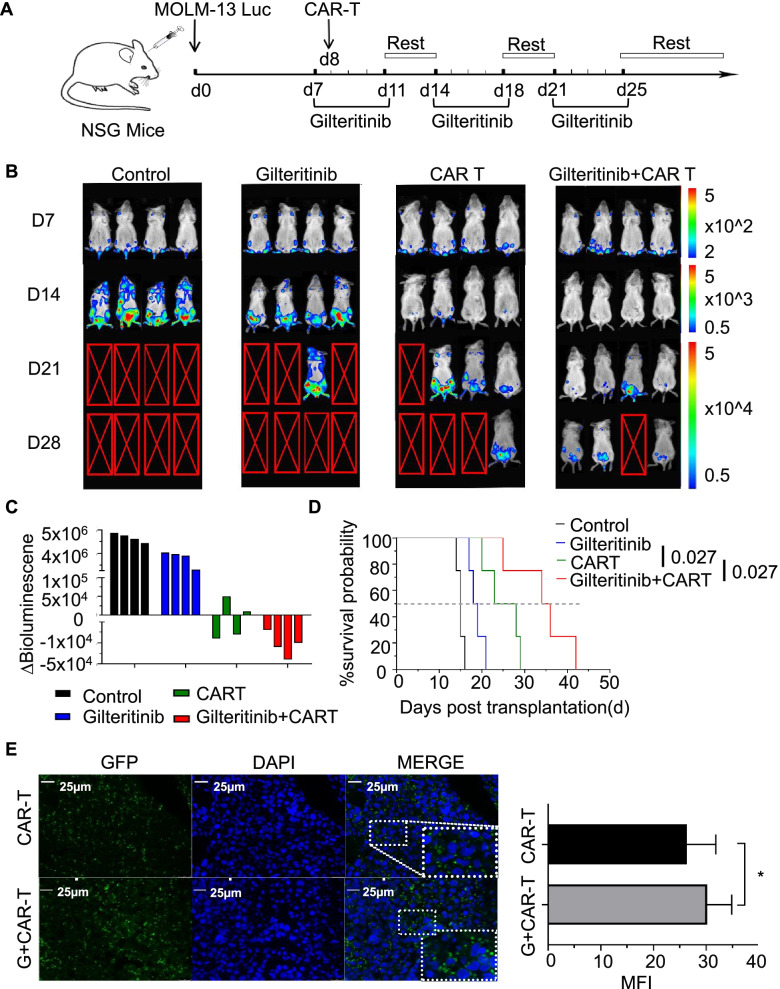
The combination of FLT3scFv/NKG2D-CAR T cells and gilteritinib act synergistically in mediating the regression of AML in a xenograft mouse model. **A** Schema of establishing the AML xenograft mouse model. NSG mice were injected with MOLM-13 luciferase-expressing cells (2 × 10^6^ cells) via the tail vein. On day 7, gilteritinib was dissolved in 4% DMSO and administered via intraperitoneal (i.p.) injection at a dose of 15 mg/kg, which was continued 5 days/week for 3 weeks in Groups 2 and 4. On day 8, a single dose of FLT3scFv/NKG2D-CAR T cells (2.5 × 10^6^ cells) was injected via the tail vein in Groups 3 and 4 (*n* = 4 mice/group). **B** Leukaemia progression was monitored by serial bioluminescent (BL) imaging using an IVIS Lumina imaging system following D-luciferin substrate administration (0.3 mg/g body weight, IP). The scale (right) shows the upper and lower BL imaging thresholds at each analysis time point (days 7, 14, 21, and 28). **C** AML burden was assessed by quantification of BL radiance obtained as photon/sec/cm2/sr in the target zone encompassing the entire body of each mouse, and the AML burden was significantly reduced in mice treated with FLT3scFv/NKG2D-CAR T cells alone and in combination with gilteritinib. The waterfall plot shows the ∆BL value (upper-increase/below-decrease) as absolute BL values obtained from each mouse between day 7 and day 14 after tumour inoculation. **D** Kaplan–Meier survival curves showed the survival of AML mice was significantly improved with CAR T cell monotherapy (*p* < 0.05 compared with gilteritinib monotherapy) and with combination therapy (*p* < 0.05 compared with CAR T cell monotherapy). The in vivo data shown are derived from two independent experiments with T cells provided from 2 different donors. **E** Immunofluorescence staining showed that the distribution of CAR T cells in the bone marrow of AML mice was significantly enhanced following combination with gilteritinib treatment. The diagram shows the MFI of GFP + CAR T cells from *n* = 3 mice in each group as assessed with ImageJ. MFI, mean fluorescence intensity. GFP, green fluorescent protein. * *p* < 0.05; ** *p* < 0.01; *** *p* < 0.001; **** *p* < 0.0001

Such a synergistic effect was also recapitulated in a xenograft mouse model established by intravenous injection of MOLM-13 luciferase-expressing cells into NSG mice. On day 7, systemic leukaemia developed, and MOLM-13 luciferase cells had heavily infiltrated the bone marrow, liver and spleen (Fig. 3B). AML-bearing mice were treated with gilteritinib, FLT3scFv/NKG2D-CAR T cells, or the combination of gilteritinib and FLT3scFv/NKG2D-CAR T cells for 3 weeks (Fig. 3A). Leukaemia progression was monitored by serial BL imaging following D-luciferin substrate administration. After one week of treatment, gilteritinib alone showed an antitumour effect, and the survival time of tumour-bearing mice were improved compared with the control treatment (median survival: 19 days vs. 15 days, *p* < 0.05) (Fig. 3D), but tumours did not decrease in size (Fig. 3B, C). CAR T cell monotherapy significantly reduced the tumour burden (Fig. 3B), and the survival time of animals in this group was significantly longer than that of animals in the control group (median survival: 24 days vs. 15 days, *p* < 0.05) and the gilteritinib group (median survival: 24 days vs. 19 days, *p* < 0.05) (Fig. 3D). The combination therapy further decreased tumour burden (Fig. 3C) and increased survival time compared to CAR T cell monotherapy (median survival: 35 days vs. 24 days, *p* < 0.05) (Fig. 3D). We also used an anti-GFP antibody to visualize the distribution of CAR T cells in the bone marrow under a fluorescence microscope. As shown in Fig. 3E, the number of cells expressing GFP was greater in mice treated with the combination therapy than in those treated with CAR T cell monotherapy. Overall, these results indicate that gilteritinib treatment can augment the effect of FLT3scFv/NKG2D-CAR T cells against AML cells and enhance the distribution of FLT3scFv/NKG2D-CAR T cells in the bone marrow of AML mice.

### Gilteritinib upregulates FLT3 and NKG2DLs in AML cells

**Fig. 4:**
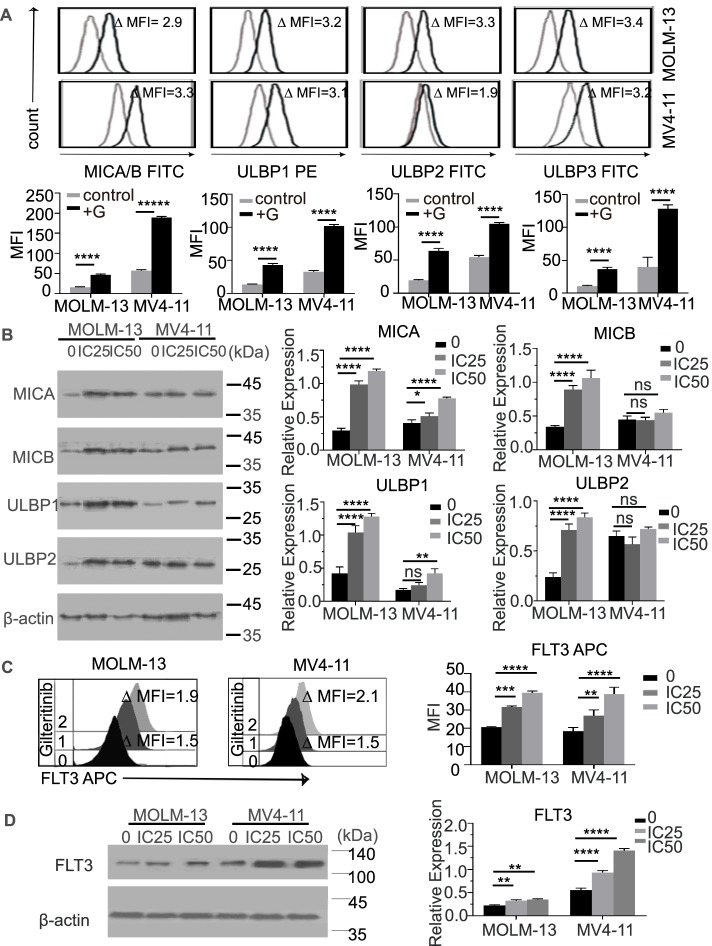
Gilteritinib upregulated the expression of NKG2DLs and FLT3 in MOLM-13 and MV4-11 cell lines. **A** Flow cytometry analysis showed that the expression of NKG2DLs (MICA/B and ULBP1/3) in MOLM-13 and MV4-11 cells was significantly upregulated with gilteritinib treatment. Histograms show NKG2DL expression on MOLM-13 cells and MV4-11 cells in the absence (grey) and presence (black) of IC25-gilteritinib for 24 h. Inset numbers show the ratio in MFI of treated/nontreated cells. **B** Western blotting (left) showed that the protein levels of all NKG2DLs in the MOLM-13 cell line were significantly increased with gilteritinib treatment, but in the MV4-11 cell line, only the MICA and ULBP1 levels were upregulated. The diagrams (right) show the relative expression as the grey intensity ratio of the NKG2DL (MICA ~ B, ULBP1 ~ 2) protein band to the internal control **(C)** Flow cytometry analysis of FLT3 expression on cell lines (MOLM-13 and MV4-11) with or without gilteritinib treatment. The histograms show FLT3 expression in MOLM-13 and MV4-11 cells treated without gilteritinib (0), 24-h IC25 gilteritinib (1), and 24-h IC50 gilteritinib (2). **D** Western blotting (left) indicated that FLT3 expression in both cell lines was significantly upregulated by gilteritinib pre-treatment. The diagrams (right) show the relative expression as the grey intensity ratio of the FLT3 protein band to the internal control. G, gilteritinib; FITC, fluorescein isothiocyanate; MFI, mean fluorescence intensity. ns, not significant. * *p* < 0.05; ** *p* < 0.01; *** *p* < 0.001; **** *p* < 0.0001

To investigate the mechanism behind the synergistic effect of gilteritinib and FLT3scFv/NKG2D-CAR T cells against AML, we investigated whether gilteritinib can affect NKG2DL and FLT3 expression. After treatment with gilteritinib at a low dose (IC_25_ of MOLM-13: 0.232 nM; IC_25_ of MV4-11: 0.66 nM), a significant increase in NKG2DL (MICA/B and ULPB1-3) expression was detected in MOLM-13 cells using flow cytometry and Western blotting (all *p* < 0.001 compared with PBS controls) (Fig. 4A-B). In MV4-11 cells, the increase in NKG2DL expression was similar to that in MOLM-13 cells detected by flow cytometry (Fig. 4A-B), but the MICB and ULBP2 expression was not significantly different between the gilteritinib and PBS treatment groups by Western blotting (*p* = ns) (Fig. 4B), indicating that gilteritinib may selectively upregulate NKG2DL expression. We also found that gilteritinib treatment led to a substantial elevation in FLT3 expression in both cell lines, as detected by flow cytometry and Western blotting (Fig. 4C-D).

**Fig. 5:**
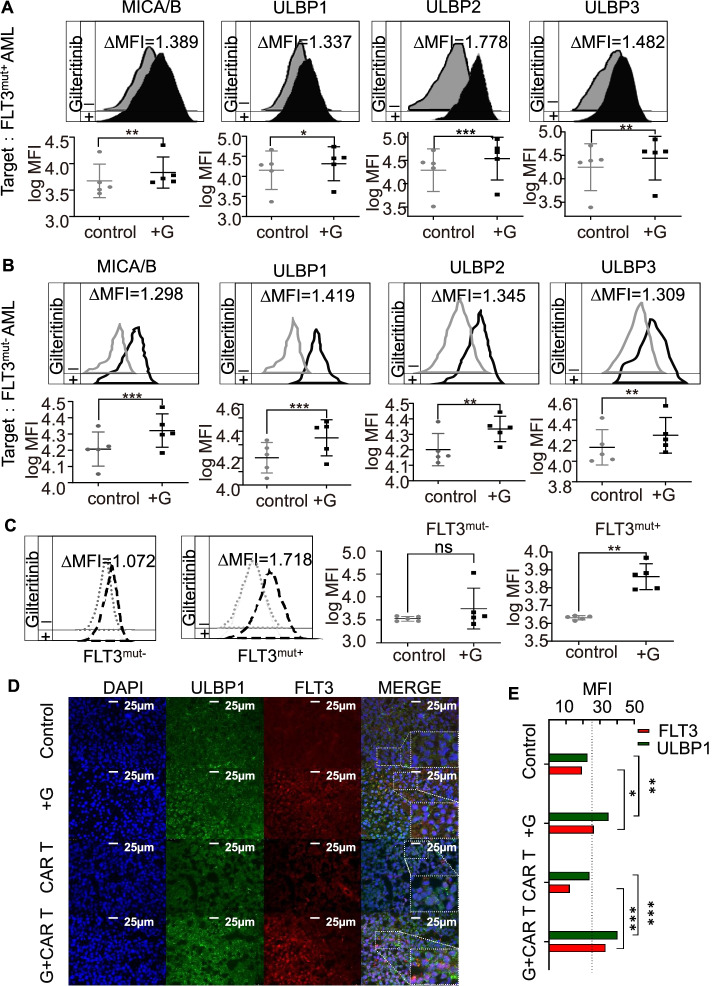
Gilteritinib upregulated the expression of NKG2DLs and FLT3 in AML cells from patients with FLT3^mut+^ and FLT3^mut−^ AML and in the bone marrow of xenograft mouse models. **A-B** Flow cytometry analysis indicated that NKG2DL expression in AML cells from patients with FLT3^mut+^ or FLT3^mut−^ AML was significantly elevated with gilteritinib treatment. Histograms show NKG2DL expression on FLT3^mut+^ or FLT3^mut−^ AML in the absence (grey) and presence (black) of gilteritinib for 24 h. Inset numbers showed the ratio in MFI of treated/non-treated cells. **C** The upregulation of FLT3 following gilteritinib treatment was only detected in cells from patients with FLT3^mut+^ AML and not in cells from patients with FLT3^mut−^ AML. Histograms show FLT3 expression on FLT3^mut+^ or FLT3^mut−^ AML in the absence (grey) and presence (black) of gilteritinib for 24 h. Inset numbers showed the ratio in MFI of treated/non-treated cells. **D** In xenograft models, bone marrow immunofluorescence staining showed the density of NKG2DL ULBP1 and FLT3 cells. **E** The diagram showed the MFIs of NKG2DL ULBP1 (green) and FLT3 (red) measured by ImageJ from *n* = 3 mice in each group. G, gilteritinib; MFI, mean fluorescence intensity. ns, not significant. * *p* < 0.05; ** *p* < 0.01; *** *p* < 0.001

To determine whether these changes are clinically relevant, we used gilteritinib to stimulate bone marrow mononuclear cells derived from patients with FLT3^mut+^ AML or FLT3^mut−^ AML. The flow cytometry results showed that following gilteritinib (IC25) treatment, the expression of NKG2DLs was significantly increased compared with that in the PBS controls in both FLT3^mut+^ (Fig. 5A) and FLT3^mut−^ AML cells (Fig. 5B). However, the upregulation of FLT3 only occurred in FLT3^mut+^ AML cells, not in FLT3^mut−^ AML cells (Fig. 5C). Elevated ULBP1 and FLT3 expression was also observed in bone marrow cells of AML-bearing mice treated with gilteritinib monotherapy or gilteritinib combined with CAR T cells (all *p* < 0.05 compared with controls) (Fig. 5D, E). These in vitro and in vivo data suggest that gilteritinib may promote the antileukaemic activities of FLT3scFv/NKG2D-CAR T cells by upregulating NKG2DL and FLT3 expression in AML cells, which enhances the AML targeting efficiency of CAR T cells. Besides, gilteritinib pretreatment enhanced the proliferation of FLT3scFv/NKG2D CAR-T cells (Supplementary Fig. 6B) and promoted FLT3scFv/NKG2D CAR-T cell-target cell conjunction formation (Supplementary Fig. 7).

### The noncanonical NF-κB2/Rel B signalling pathway mediates gilteritinib-induced NKG2DL upregulation

**Fig. 6:**
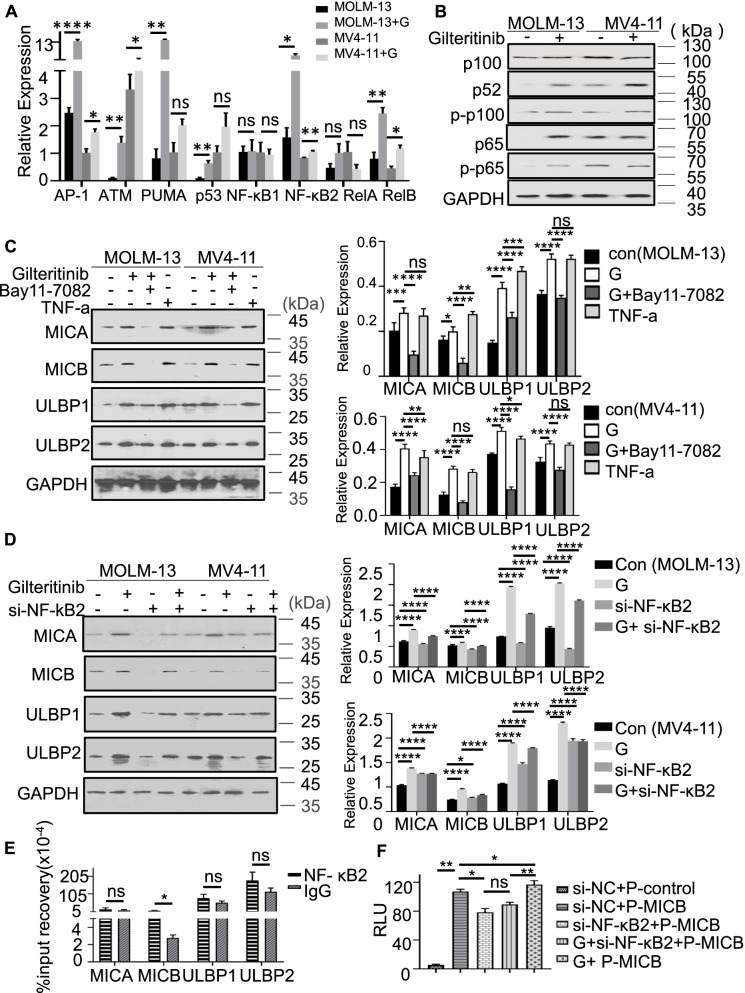
Gilteritinib upregulated NKG2DL expression via the noncanonical NF-κB2/Rel B signalling pathway.** A** MOLM-13 and MV4-11 cells were treated with IC25 gilteritinib for 24 h followed by real-time PCR. The diagrams showed that gilteritinib elevated the mRNA expression of NF-κB upstream and downstream target genes. **B** Western blotting on the MOLM-13 and MV4-11 cells pre-treated with IC25 gilteritinib for 24 h. The protein levels of p100, p52, and p65 and the phosphorylation of p100 and p65 in MOLM-13 and MV4-11 cells were significantly increased with gilteritinib treatment. **C** MOLM-13 and MV4-11 cells were treated with 10 nM TNF-a for 24 h or 2.5uM BAY11-7082 for 12 h following IC25-gilteritinib for 24 h. The gilteritinib-induced upregulation of NKG2DLs in AML cells was similar to that induced by TNF-α but was significantly suppressed by the NF-κB inhibitor BAY 11–7082 at the protein level. **D** MOLM-13 and MV4-11 cells were transfected with si-NF-kB2 for 72 h and then incubated with IC25-gilteritinib for 24 h. Silencing NF-κB2 downregulated gilteritinib-induced NKG2DL expression in AML cells, indicating that gilteritinib regulated NKG2DL expression via the noncanonical NF-κB2/Rel B signalling pathway. **E** ChIP analysis showed that NF-κB2 binds to the MICB promoter. **F** In the absence or presence of 72-h siRNA (siRNA-NC or siRNA- NF-κB2) transfection, MOLM-13 cells were transfected with luciferase reporter vectors (PGL3-vector or PGL3-MICB) using Lipofectamine 3000. MOLM-13 cells were treated with or without IC25-gilteritinib for 24 h, and then the luciferase activity was detected by a dual luciferase assay system. The histogram showed that RLU represents the luciferase activity normalized to pRL-TK luciferase activity for each group. Luciferase assays showed that NF-κB2 was able to activate the transcription of MICB. The data presented are derived from the results of three independent experiments. G, gilteritinib; Con, control; RLUs, relative light units; P-control, pGL3-control vector; ns, not significant. * *p* < 0˜.05; ** *p* < 0.01; *** *p* < 0.001; **** *p* < 0.0001

To evaluate the mechanism underlying gilteritinib-induced NKG2DL upregulation, we treated MOLM-13 and MV4-11 cells with gilteritinib and then assessed a wide range of genes related to apoptosis, DNA damage and the NF-κB family. Interestingly, we found that gilteritinib treatment significantly upregulated the expression of ATM, p53, PUMA, AP-1, NF-κB2 and Rel B (all *p* < 0.05 compared with the control treatment) but not NF-κB1 and Rel A (Fig. 6A). The changes were prominent in MOLM-13 cells. The protein levels of p100, NF-κB2 and phosphorylated p100 were also significantly elevated in both MOLM-13 and MV4-11 cell lines after gilteritinib treatment (Fig. 6B). We therefore hypothesized that gilteritinib might upregulate NKG2DLs through an interaction with the noncanonical NF-κB2/Rel B signalling pathway. To confirm this, AML cells were cultured with gilteritinib, TNFα, and gilteritinib in combination with BAY 11–7082 (an NF-κB inhibitor that inhibits TNFα-induced IκBα phosphorylation). We found that gilteritinib induced a significant increase in NKG2DL protein levels, which was similar to that induced by TNFα. This elevation was blocked by BAY 11–7082 treatment (Fig. 6C). Furthermore, when AML cells were cocultured with gilteritinib and NF-κB2 siRNA, the protein levels of NKG2DLs were significantly lower than those in cells incubated with gilteritinib alone (Fig. 6D), indicating that silencing NF-κB2 hampers the gilteritinib-induced upregulation of NKG2DLs at the protein level.

To ascertain the transcriptional role of NF-κB2 in gilteritinib-induced NKG2DL expression in MOLM-13 cells, we performed quantitative chromatin immunoprecipitation (ChIP) assay and found that the binding of NF-κB2 at the promoter regions of MICB was considerably reduced (Fig. 6E), indicating that NF-κB2 can modulate MICB transcription (Supplementary Fig. 5). We did not detect any interaction between NF-κB2 and other NKG2DLs, including MICA and ULBPs, in MOLM-13 cells. To confirm that NF-κB2 can directly and positively regulate the transcription of MICB, we successfully constructed a PGL-MICB plasmid expressing fluorescein (Si-NC + P-MICB vs. Si-NC + P-control, *p* < 0.05). The results showed that when NF-kB2 was silenced, the transcriptional fluorescence activity of PGL3-MICB was significantly reduced (Si-NF-kB2 + P-MICB vs. Si-NC + P-MICB, *p* < 0.05), suggesting that NF-κB2 can positively regulate the transcription of MICB. The combined treatment with gilteritinib significantly increased the transcriptional fluorescence activity of PGL-MICB (G + P-MICB vs. Si-NC + P-MICB, *p* < 0.05) when NF-kB2 was not silenced. When NF-kB2 was silenced, gilteritinib reversed the transcriptional fluorescence activity of Si-NF-kB2 + P-MICB (G + Si-NF-kB2 + P-MICB vs. Si-NF-kB2 + P-MICB,* p* > 0.05) (Fig. 6F). These results suggest that gilteritinib positively regulated the transcription of MICB through NF-kB2. Overall, gilteritinib can activate the noncanonical NF-kB2/Rel B signalling pathway, upregulate NF-kB2 and promote the binding of NF-kB2 to the MICB promoter region to activate the transcription of MICB, consequently increasing the expression of NKG2DLs.

### The effect of combination of FLT3scFv/NKG2D-CAR T cells and gilteritinib on CD34 + HSCs

Bone marrow damage is one of the most common side effects of conventional cancer treatment. We therefore determined whether FLT3scFv/NKG2D-CAR T cells could target normal haematopoietic stem cells (HSCs) as an on-target off-tumour effect. HSCs were obtained from neonatal cord blood (3 donors), and CD34 + HSCs accounted for 94.5 ± 2.3% of cells (Supplementary Fig. 1A). After incubation with gilteritinib, the expression of FLT3 and NKG2DLs in HSCs was unchanged compared to that in the controls (Supplementary Fig. 1B-C). This result indicated that gilteritinib did not affect FLT3 and NKG2DL expression in normal HSCs. Flow cytometry-based cytotoxicity assays showed that FLT3scFv/NKG2D-CAR T cells lysed approximately 7% of normal HSCs at an effector:target (E:T) ratio of 1:1, 20% of normal HSCs at E:T = 10:1, and 23% of normal HSCs at E:T = 20:1 (all *p* < 0.05 compared with UTD controls). Notably, combined treatment with gilteritinib did not cause more cytotoxicity to normal HSCs than CAR T cell monotherapy (Supplementary Fig. 1D). Colony formation assays showed a significant reduction in GEMM, CFU-G/M and BFU-E colonies in HSCs cultured with CAR T cells with or without gilteritinib (all *p* < 0.05 compared with UTD cells) (Supplementary Fig. 1E). These results suggest that FLT3scFv/NKG2D-CAR T cells have certain cytotoxic effects against normal HSCs that are not affected by additional gilteritinib treatment (Supplementary Fig. 1).

We also assessed the effect of gilteritinib on FLT3scFv/NKG2D-CAR T cells. Flow cytometric analysis showed that gilteritinib had no significant cytotoxicity against FLT3scFv/NKG2D-CAR T cells after 24 h of treatment (Supplementary Fig. 2A, Supplementary Fig. 6A). The expression of FLT3scFv and NKG2D in CAR T cells was not affected by gilteritinib treatment (Supplementary Fig. 2B). The levels of IL-2, IL-6 and GM-CSF released by FLT3scFv/NKG2D CAR-T cells were also significantly higher than those released by UTD cells, while gilteritinib did not enhanced such inflammatory cytokine release (Supplementary Fig. 9).

## Discussion

Relapsed/refractory FLT3^mut+^ AML has a poor response to most conventional treatments and FLT3 inhibitors. The efficacy of single-target CAR T cell therapy is inconclusive. Exploring new modalities is the key to improving the OS of patients with this disease. Recently, combined therapy and dual-target CAR T cell therapies have attracted much attention. Studies have suggested that FLT3 and NKG2DLs as AML-specific antigens due to their high expression in AML cells but low or no expression in normal cells [26, 27]. In line with these findings, we also detected selective expression of NKG2DLs and FLT3 in bone marrow mononuclear cells from AML patients. We therefore generated CAR T cells based on a new CAR construct comprising the extracellular region of the human NKG2D receptor and FLT3scFv to provide a therapeutic option for FLT3^mut+^ AML patients. Our data demonstrated that bispecific FLT3scFv/NKG2D-CAR T cells effectively had binding capacity and cytotoxicity against FLT_3_^mut+^ AML cells and significantly prolonged the survival of the mice with AML established by MOLM-13 cell implantation. Encouragingly, the bispecific CAR T cells eliminated not only primary FLT_3_^mut+^ AML blasts but also FLT_3_^mut−^ AML blasts, although the treatment effect on the FLT_3_^mut+^ AML blasts was more profound. The tumour-killing efficacy of the CAR T cells improved dramatically when they were administered in combination with gilteritinib both in vitro and in vivo.

To our knowledge, this is the first attempt to generate bispecific CAR T cells concurrently targeting NKG2DLs and FLT3. Our preclinical data indicate therapeutic efficacy in the form of better survival of tumour-bearing mice following treatment. Bispecific CAR T cells recognize two tumour antigens specific to AML, thereby creating synergistic effects to target all AML cells expressing either NKG2DLs or FLT3. This notion is particularly important for two critical factors in AML management. The first is FLT3-ITD AML, which has poor prognosis and unfavourable therapeutic outcomes [1, 28–30]. Although some combined therapies or monotherapies employing a new generation of FLT3 inhibitors have proven to be effective in treating FLT3-ITD AML, subsequent drug resistance poses a substantial challenge [31, 32]. The underlying mechanisms include on-target secondary mutations such as F691L mutations that weaken FLT3 inhibition [33], persistent activity of the FLT3/MAPK pathway that promotes leukemic blast survival [34], increased FLT3 ligand levels [35] and upregulation of Ras/MAPK signalling independent of the FLT3 receptor [36] in the bone marrow microenvironment. Theoretically, the NKG2D compartment of our bispecific CAR T cells can disrupt the oncogenic signalling initiated by FLT3 and other molecules under such circumstances, thereby preventing resistance to FLT3 targeting. This idea was confirmed by the significant eradication of AML cells from FLT3-ITD patients by FLT3scFv/NKG2D-CAR T cells in this study. Another factor is tumour escape mediated by NKG2D/NKG2DLs [21]. Malignant cells develop diverse strategies to avoid NKG2D-mediated cytolysis, one of which is the release of soluble NKG2DLs from the tumour cell surface into the serum [37]. These soluble molecules can bind to NKG2D receptors on NK cells, consequently impairing the anti-leukaemia capacity of NK cells by reducing the recognition of AML cells by NK cells [38]. This notion may also apply to NKG2D-CAR T cells and reduce the efficacy of CAR T cell therapy. Therefore, utilizing multiple CARs to target different AML-associated antigens can counterbalance the disadvantages of single targeting. Furthermore, AML blasts may also lose surface expression of a particular tumour antigen under the pressure of CAR T cell therapy [13, 39]. With the use of bispecific CAR T cells, such therapy-induced loss of one specific target can be compensated for by another component. Therefore, FLT3scFv/NKG2D-CAR T cells not only facilitate tumour-killing activities but also prevent the emergence of tumour resistance using multimodal mechanisms.

We observed that the therapeutic effects of CAR T cells improved greatly when AML cells were pre-treated with gilteritinib. Gilteritinib selectively upregulated the expression of NKG2DLs (MICA/B and ULBPs), thereby increasing the tumour-killing capacity of CAR T cells. This result is in line with our previous report that NKG2DLs are upregulated by the FLT3 inhibitor sunitinib in nasopharyngeal carcinoma and haematoma cells [23]. For the first time, we confirmed the NF-κB2 dependency of gilteritinib-induced NKG2DL upregulation. Using ChIP and luciferase assays, we identified the promoter regions of MICB (NKG2DL) that interact with NF-κB2. Our findings add another clue to existing knowledge about the signalling pathways involved in NKG2DL upregulation in AML cells [21]. However, the upregulation of NKG2DL expression induced by gilteritinib may involve multiple mechanisms. p53 and NF-κB act as transcription factors of MICA and ULBP2, respectively [40, 41]. Upregulation of these molecules was also observed among gilteritinib-pretreated AML cells. Beyond the upregulation of NKG2DLs, FLT3 upregulation in FLT_3_^mut+^ AML cells following gilteritinib treatment was also found. Other researchers have reported similar effects of other FLT3 inhibitors, such as lestaurtinib [42] and crenolanib [16], on FLT3 expression in clinical and preclinical AML settings. Further exploration in this regard is desirable. Taken together, the data show that the immune-sensitizing effect of gilteritinib on AML cells can be potentiated when it is administered in combination with FLT3scFv/NKG2D-CAR T cell therapy. Such combinatorial treatment promotes the specific targeting of AML cells via multiple targets to achieve complete remission of AML. In addition, using gilteritinib as an induction and bridging treatment during the period of T cell manufacturing can provide more patients with opportunities for CAR T cell therapy. For the first time, our data provide proof of concept for the combination of FLT3scFv/NKG2D-CAR T cells and gilteritinib as a synergistic therapy for relapsed/refractory FLT_3_^mut+^ AML.

In the current study, we also evaluated the effect of CAR T cell therapy in normal HSCs with different doses of CAR T cells. We found that FLT3scFv/NKG2D-CAR T cells lysed 7–23% of HSCs in a dose-dependent manner. Previously, a phase 1 clinical trial revealed no grade 3 or above adverse events related to NKG2D CAR T cell therapy in patients with relapsed/refractory AML [43], indicating that it is safe targeting NKG2DLs in AML patients. Clinical data regarding the safety of FLT3-CAR T cells are currently not available, but a nonclinical safety assessment demonstrated that FLT3-CAR T cells only affect a small percentage of normal haematopoietic stem and progenitor cells [44], which is in agreement with our findings. In contrast, other studies have reported that FLT3-CAR T cells do not deplete HSCs and that HSC differentiation is preserved [17]. Studies also have found that another side effect of FLT3scFv/NKG2D CAR-T cell therapy may be cytokine release syndrome (CRS), while this adverse effect did not enhanced by gilteritinib pretreatment in vitro. Moreover, we did not find a significant cytotoxic effect of gilteritinib on FLT3scFv/NKG2D-CAR T cells. Therefore, using the combination of gilteritinib and FLT3scFv/NKG2D-CAR T cell for AML treatment is feasible.

## Conclusions

Bispecific FLT3scFv/NKG2D-CAR T cells can effectively kill AML cells. This tumour-killing capacity can be synergistically improved by the specific FLT3 inhibitor gilteritinib via upregulation of FLT3 and NKG2DL expression in AML cells. Gilteritinib-induced NKG2DL upregulation is NF-κB2 dependent. The present study provides a rational for the combined use of gilteritinib and FLT3scFv/NKG2D-CAR T cells as therapy for relapsed/refractory FLT3^mut+^ AML.

## Supplementary information

**Additional file 1:** **Figure S1.** Cytotoxicity of FLT3scFv/NKG2D-CAR T cells to normal human hematopoietic stem cells (HSCs)

**Additional file 2:** **Figure S2.** The effect of gilteritinib on FLT3scFv/NKG2D CAR T.

**Additional file 3: Figure S3.** FLT3 inhibitor Gilteritinib inhibits proliferation and induced apotosis in FLT3^mut+^AML cell lines.

**Additional file 4:** **Figure S4.** FLT3scFv/NKG2D-CAR T cells show certain cytotoxicity aginst FLT3^mut-^ AML cell lines in vitro

**Additional file 5:** **Figure S5. **The transcriptional role of NF-κB2 in NKG2DL expression

**Additional file 6:** **Figure S6.** Gilteritinib pretreatment enhanced the proliferation of FLT3scFv/NKG2D CAR-T cells

**Additional file 7:** **Figure S7.** Gilteritinib pretreatment promotes FLT3scFv/NKG2D CAR-T cell-target cell conjunction formation

**Additional file 8:** **Figure S8.** The properties of FLT3scFv/NKG2D CAR T cells as compared to UTD cells.

**Additional file 9: Figure S9. **Cytokine secretion of UTD cells and FLT3scFv/NKG2D CAR-T after coculture with target cells.

**Additional file 10:** Supplementary methods

**Additional file 11:** **Table S1.** The sequences of FLT3scFv and NKG2D utilised in CAR

**Additional file 12: Table S2.** Clinical characteristics of patients with acute myeloid leukaemia (AML)

**Additional file 13:** **Table S3.** Antibodies used for flow cytometry and Western blot

**Additional file 14:** **Table S4.** The sequences of forward/reverse primers for target genes

**Additional file 15:** **Table S5.** The sequences of forward/reverse primers for chromatin immunoprecipitation (ChIP) assay

## Data Availability

The datasets used and/or analysed during the current study are available from the corresponding author on reasonable request.

## Abbreviations

AML: Acute myeloid leukaemia
ANOVA: Analysis of variance
AP-1: Activator protein 1
ATM: Ataxia-telangiesctasia mutated
BL: Bioluminescene
CAR: Chimeric antigen receptor
DMSO: Dimethyl sulfoxide
FBS: Fetal bovine serum
FDA: Food and Drug Administration
FLT3: FMS-like tyrosine kinase 3
GFP: Green fluorescent protein
HSCT: Hematopoietic stem cell transplantation
IL-2: Interleukin 2
ITD: Internal tandem duplication
IFNγ: Interferon gamma
MOI: Multiplicity of infection
NF-κB: Nuclear factor-kappa-B
NKG2D: Natural killer group 2 member D
NKG2DLs: NKG2D ligands
Ns: Not significant
UTD: Un-transduced T cells
PBS: Phosphate buffered saline
PBMCs: Peripheral blood mononuclear cells
PGL3: Paragangliomas-3
scFv: Single chain fragment variable
TKD: Tyrosine kinase domain
UTD: Un-transduced
TP53: Tumor protein p53
BCL2: B-cell lymphoma-2
MCL1: MCL1 apoptosis regulator
PUMA: p53 upregulated modulator of apoptosis
ATM: Ataxia-telangiectasia mutated proteins
ATR: ATM- and RAD3-related
NFKB: Nuclear factor kappa B subunit
REL: REL proto-oncogene, NF-kB subunit
GAPDH: Glyceraldehyde-3-phosphate dehydrogenase
NKG2D: Natural Killer group 2 member D
MIC: major histocompatibility complex class I chain-related gene
ULBP: UL16 binding protein
