# Correction: A novel approach for relapsed/refractory FLT3^mut+^acute myeloid leukaemia: synergistic effect of the combination of bispecific FLT3scFv/NKG2D-CAR T cells and gilteritinib

**DOI:** 10.1186/s12943-022-01566-0

**Published:** 2022-06-23

**Authors:** Ke-xin Li, Hui-yang Wu, Wan-ying Pan, Meng-qi Guo, De-zhi Qiu, Yan-jie He, Yu-hua Li, Dong-Hua Yang, Yu-xian Huang

**Affiliations:** 1 Department of Hematology, Zhujiang Hospital, Southern Medical University, Guangzhou, 510282 Guangdong China; 2 Department of Pharmaceutical Sciences, College of Pharmacy and Health Sciences, St. John’s University, Queens, NY 11439 USA

**Correction: Mol Cancer 21, 66 (2022)**

**https://doi.org/10.1186/s12943-022-01541-9**

Following publication of the original article [1], the authors identified minor errors in Figs. 3, 4, 5 and Additional file 5: Figure S5, and Additional file 14: Table S4; specifically:

- Fig. 3B: 'Control' panel was originally a duplicate of the Giliteritinib panel; this has been replaced by the correct images
- Fig. 4A: The ordinate label was incorrectly listed as 'FSC'; the correct listing is 'count'
- Fig. 4B and Fig. 4C: the column color of the statistical histograms in did not originally correspond to the legend color; this has been corrected
- Fig. 5D: incorrect images were used for the control group and +G group (rows 1 and 2); these have been replaced by the correct images
- Additional file 5: Figure S5A: incorrect image used for the MICB band; this has been replaced with the correct image
- Additional file 14: Table S4: The sequences of forward/reverse primers for Rel A, Rel B and c-Rel were found to be erroneously completed; this has been corrected

The corrected figures and table are given here. The correction does not have any effect on the final conclusions of the paper. The original article has been corrected.

**Fig. 3:**
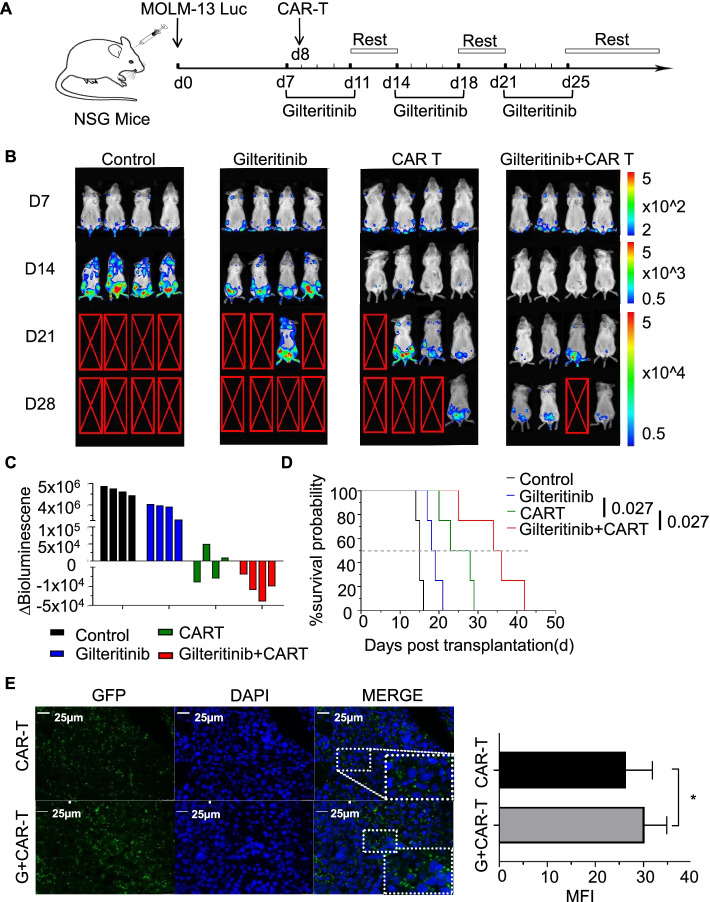
The combination of FLT3scFv/NKG2D-CAR T cells and gilteritinib act synergistically on mediating the regression of AML in a xenograft mouse model. **A** Schema of establishing the AML xenograft mouse model. NSG mice were injected with MOLM-13 luciferase-expressing cells (2 × 10^6^ cells) via the tail vein. On day 7, gilteritinib was dissolved in 4% DMSO and administered via intraperitoneal (i.p.) injection at a dose of 15 mg/kg, which was continued 5 days/week for 3 weeks in Groups 2 and 4. On day 8, a single dose of FLT3scFv/NKG2D-CAR T cells (2.5 × 10^6^ cells) was injected via the tail vein in Groups 3 and 4 (*n* = 4 mice/group). **B** Leukaemia progression was monitored by serial bioluminescent (BL) imaging using an IVIS Lumina imaging system following D-luciferin substrate administration (0.3 mg/g body weight, IP). The scale (right) shows the upper and lower BL imaging thresholds at each analysis time point (days 7, 14, 21, and 28). **C** AML burden was assessed by quantification of BL radiance obtained as photon/sec/cm2/sr in the target zone encompassing the entire body of each mouse, and the AML burden was significantly reduced in mice treated with FLT3scFv/NKG2D-CAR T cells alone and in combination with gilteritinib. The waterfall plot shows the ∆BL value (upper-increase/below-decrease) as absolute BL values obtained from each mouse between day 7 and day 14 after tumour inoculation. **D** Kaplan–Meier survival curves showed the survival of AML mice was significantly improved with CAR T cell monotherapy (*p* < 0.05 compared with gilteritinib monotherapy) and with combination therapy (*p* < 0.05 compared with CAR T cell monotherapy). The in vivo data shown are derived from two independent experiments with T cells provided from 2 different donors. **E** Immunofluorescence staining showed that the distribution of CAR T cells in the bone marrow of AML mice was significantly enhanced following combination with gilteritinib treatment. The diagram shows the MFI of GFP + CAR T cells from *n* = 3 mice in each group as assessed with ImageJ. MFI, mean fluorescence intensity. GFP, green fluorescent protein. * *p* < 0.05; ** *p* < 0.01; *** *p* < 0.001; **** *p* < 0.0001

**Fig. 4:**
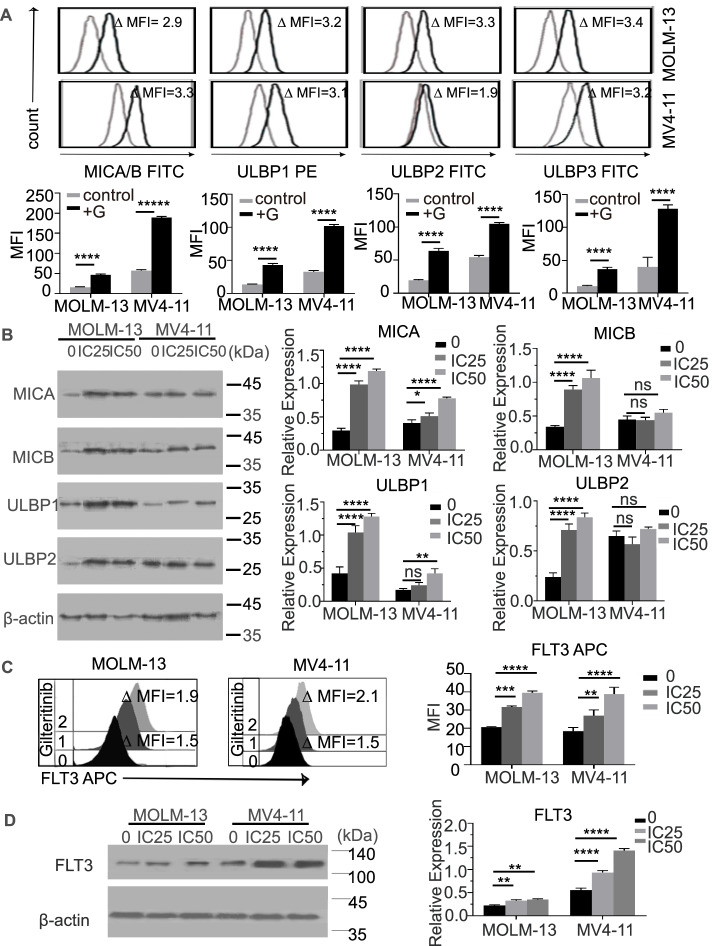
Gilteritinib upregulated the expression of NKG2DLs and FLT3 in MOLM-13 and MV4-11 cell lines. **A** Flow cytometry analysis showed that the expression of NKG2DLs (MICA/B and ULBP1/3) in MOLM-13 and MV4-11 cells was significantly upregulated with gilteritinib treatment. Histograms show NKG2DL expression on MOLM-13 cells and MV4-11 cells in the absence (grey) and presence (black) of IC25-gilteritinib for 24 h. Inset numbers show the ratio in MFI of treated/nontreated cells. **B** Western blotting (left) showed that the protein levels of all NKG2DLs in the MOLM-13 cell line were significantly increased with gilteritinib treatment, but in the MV4-11 cell line, only the MICA and ULBP1 levels were upregulated. The diagrams (right) show the relative expression as the grey intensity ratio of the NKG2DL (MICA ~ B, ULBP1 ~ 2) protein band to the internal control **(C)** Flow cytometry analysis of FLT3 expression on cell lines (MOLM-13 and MV4-11) with or without gilteritinib treatment. The histograms show FLT3 expression in MOLM-13 and MV4-11 cells treated without gilteritinib (0), 24-h IC25 gilteritinib (1), and 24-h IC50 gilteritinib (2). **D** Western blotting (left) indicated that FLT3 expression in both cell lines was significantly upregulated by gilteritinib pre-treatment. The diagrams (right) show the relative expression as the grey intensity ratio of the FLT3 protein band to the internal control. G, gilteritinib; FITC, fluorescein isothiocyanate; MFI, mean fluorescence intensity. ns, not significant. * *p* < 0.05; ** *p* < 0.01; *** *p* < 0.001; **** *p* < 0.0001

**Fig. 5:**
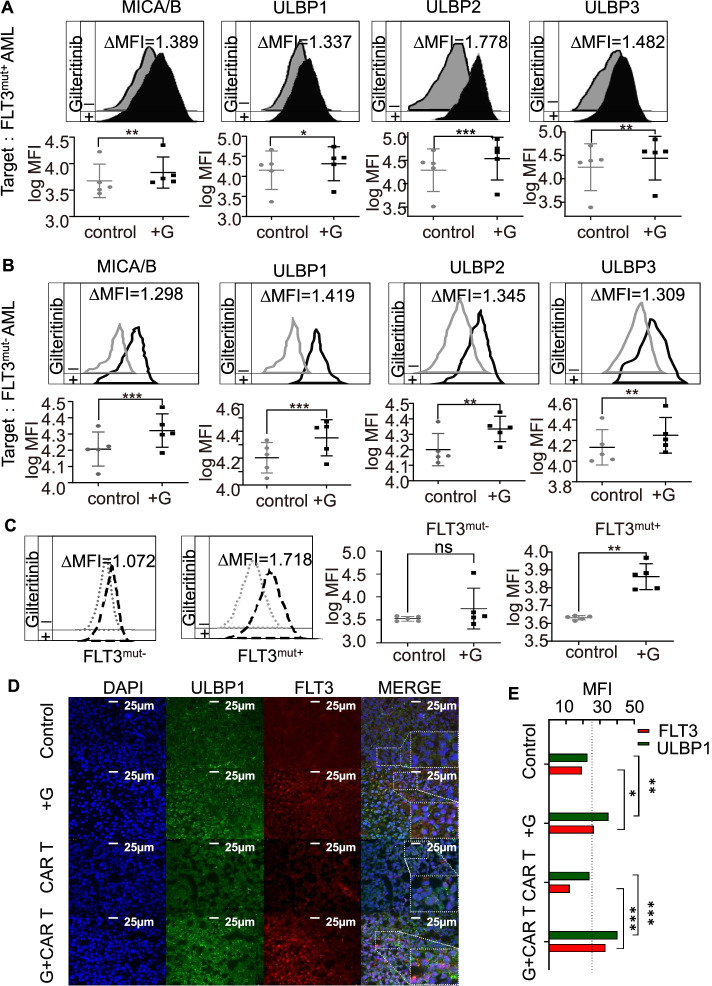
Gilteritinib upregulated the expression of NKG2DLs and FLT3 in AML cells from patients with FLT3^mut+^ and FLT3^mut−^ AML and in the bone marrow of xenograft mouse models. **A-B** Flow cytometry analysis indicated that NKG2DL expression in AML cells from patients with FLT3^mut+^ or FLT3^mut−^ AML was significantly elevated with gilteritinib treatment. Histograms show NKG2DL expression on FLT3^mut+^ or FLT3^mut−^ AML in the absence (grey) and presence (black) of gilteritinib for 24 h. Inset numbers showed the ratio in MFI of treated/non-treated cells. **C** The upregulation of FLT3 following gilteritinib treatment was only detected in cells from patients with FLT3^mut+^ AML and not in cells from patients with FLT3^mut−^ AML. Histograms show FLT3 expression on FLT3^mut+^ or FLT3^mut−^ AML in the absence (grey) and presence (black) of gilteritinib for 24 h. Inset numbers showed the ratio in MFI of treated/non-treated cells. **D** In xenograft models, bone marrow immunofluorescence staining showed the density of NKG2DL ULBP1 and FLT3 cells. **E** The diagram showed the MFIs of NKG2DL ULBP1 (green) and FLT3 (red) measured by ImageJ from *n* = 3 mice in each group. G, gilteritinib; MFI, mean fluorescence intensity. ns, not significant. * *p* < 0.05; ** *p* < 0.01; *** *p* < 0.001

## Supplementary Information

**Additional file 5: Figure S5A.** The transcriptional role of NF-κB2 in NKG2DL expression.

**Additional file 14: Table S4.** The sequences of forward/reverse primers for target genes.
