# Supplementary material for: A novel approach for relapsed/refractory FLT3mut+ acute myeloid leukaemia: synergistic effect of the combination of bispecific FLT3scFv/NKG2D-CAR T cells and gilteritinib
Source: Mol Cancer. 2022 Mar 4;21:66. doi: 10.1186/s12943-022-01541-9 (PMC8896098; doi:10.1186/s12943-022-01541-9)
Supplement: Supplementary file 1 — Additional file 1: Figure S1. Cytotoxicity of FLT3scFv/NKG2D-CAR T cells to normal human hematopoietic stem cells (HSCs) [file 12943_2022_1541_MOESM1_ESM.pptx]

## Slide 1
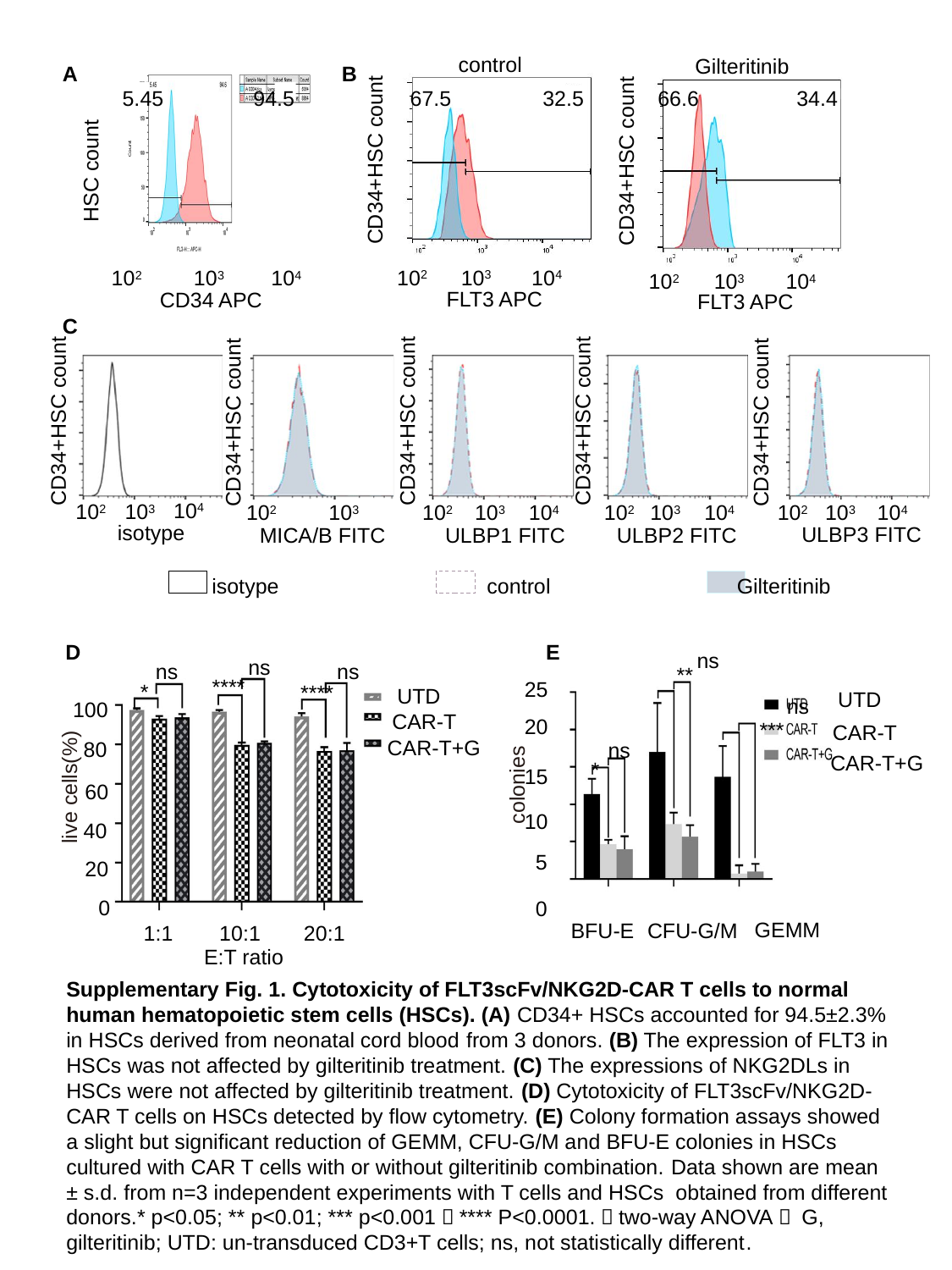

control
67.5
32.5
CD34+HSC count
102
103
104
FLT3 APC
Gilteritinib
66.6
34.4
CD34+HSC count
102
103
104
FLT3 APC
A
B
5.45
94.5
HSC count
102
103
104
CD34 APC
C
CD34+HSC count
CD34+HSC count
CD34+HSC count
CD34+HSC count
CD34+HSC count
102
103
104
102
103
104
104
102
103
102
103
isotype
ULBP3 FITC
ULBP1 FITC
MICA/B FITC
ULBP2 FITC
102
103
104
isotype
control
Gilteritinib
D
E
ns
**
UTD
ns
***
CAR-T
ns
CAR-T+G
*
colonies
GEMM
CFU-G/M
BFU-E
25
20
15
10
5
0
ns
ns
*
100
CAR-T
CAR-T+G
80
live cells(%)
60
40
20
0
1:1
10:1
20:1
E:T ratio
ns
****
****
UTD
Supplementary Fig. 1. Cytotoxicity of FLT3scFv/NKG2D-CAR T cells to normal human hematopoietic stem cells (HSCs). (A) CD34+ HSCs accounted for 94.5±2.3% in HSCs derived from neonatal cord blood from 3 donors. (B) The expression of FLT3 in HSCs was not affected by gilteritinib treatment. (C) The expressions of NKG2DLs in HSCs were not affected by gilteritinib treatment. (D) Cytotoxicity of FLT3scFv/NKG2D-CAR T cells on HSCs detected by flow cytometry. (E) Colony formation assays showed a slight but significant reduction of GEMM, CFU-G/M and BFU-E colonies in HSCs cultured with CAR T cells with or without gilteritinib combination. Data shown are mean ± s.d. from n=3 independent experiments with T cells and HSCs obtained from different donors.* p<0.05; ** p<0.01; *** p<0.001；**** P<0.0001.（two-way ANOVA） G, gilteritinib; UTD: un-transduced CD3+T cells; ns, not statistically different.
