# Supplementary material for: A novel approach for relapsed/refractory FLT3mut+ acute myeloid leukaemia: synergistic effect of the combination of bispecific FLT3scFv/NKG2D-CAR T cells and gilteritinib
Source: Mol Cancer. 2022 Mar 4;21:66. doi: 10.1186/s12943-022-01541-9 (PMC8896098; doi:10.1186/s12943-022-01541-9)
Supplement: Supplementary file 2 — Additional file 2: Figure S2. The effect of gilteritinib on FLT3scFv/NKG2D CAR T. [file 12943_2022_1541_MOESM2_ESM.pptx]

## Slide 1
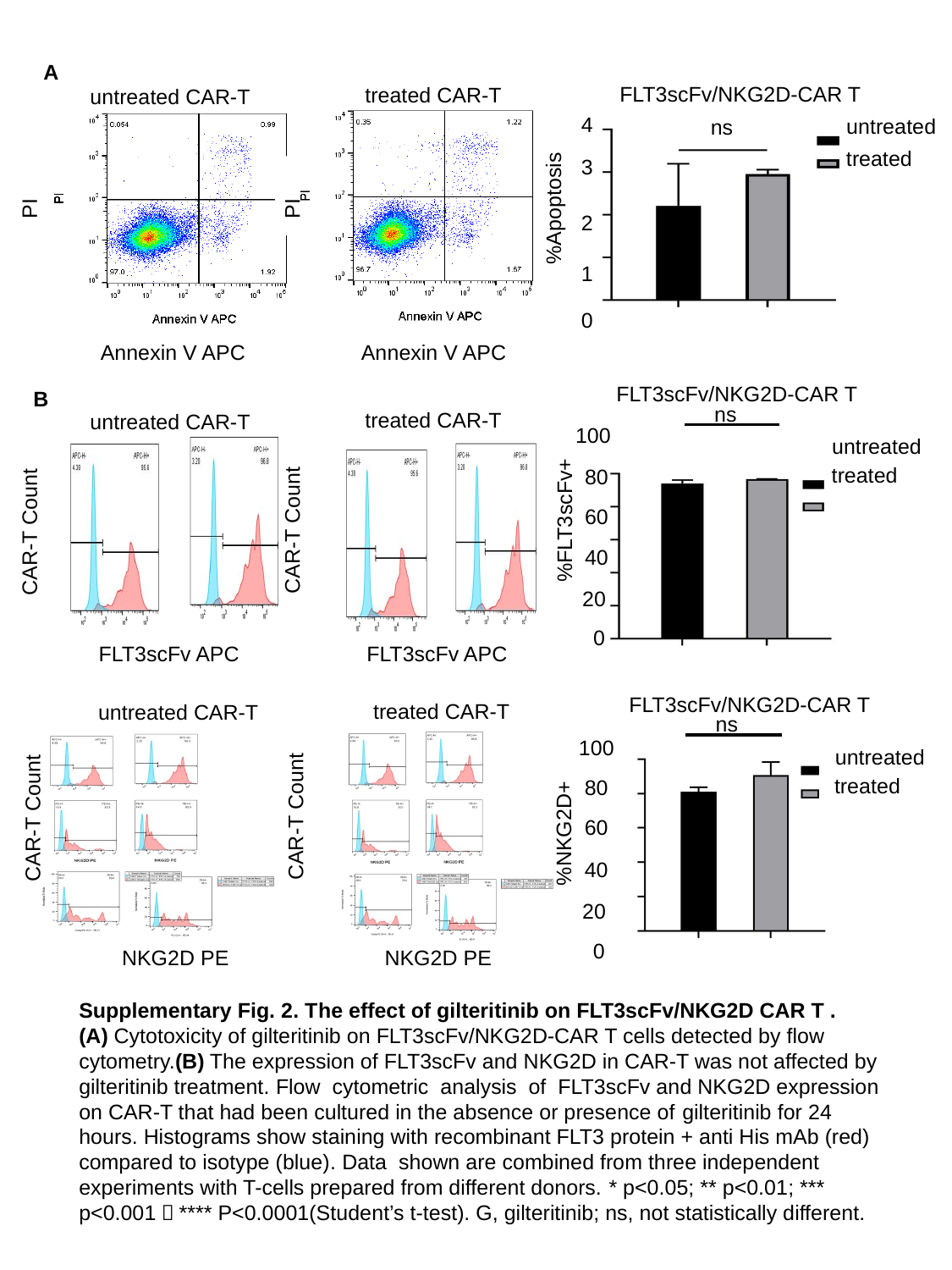

A
FLT3scFv/NKG2D-CAR T
4
untreated
ns
treated
3
%Apoptosis
2
1
0
treated CAR-T
untreated CAR-T
PI
PI
Annexin V APC
Annexin V APC
FLT3scFv/NKG2D-CAR T
ns
100
untreated
treated
80
%FLT3scFv+
60
40
20
0
B
treated CAR-T
untreated CAR-T
CAR-T Count
CAR-T Count
FLT3scFv APC
FLT3scFv APC
FLT3scFv/NKG2D-CAR T
ns
100
untreated
treated
80
%NKG2D+
60
40
20
0
treated CAR-T
untreated CAR-T
CAR-T Count
CAR-T Count
NKG2D PE
NKG2D PE
Supplementary Fig. 2. The effect of gilteritinib on FLT3scFv/NKG2D CAR T .
(A) Cytotoxicity of gilteritinib on FLT3scFv/NKG2D-CAR T cells detected by flow cytometry.(B) The expression of FLT3scFv and NKG2D in CAR-T was not affected by gilteritinib treatment. Flow cytometric analysis of FLT3scFv and NKG2D expression on CAR-T that had been cultured in the absence or presence of gilteritinib for 24 hours. Histograms show staining with recombinant FLT3 protein + anti His mAb (red) compared to isotype (blue). Data shown are combined from three independent experiments with T-cells prepared from different donors. * p<0.05; ** p<0.01; *** p<0.001；**** P<0.0001(Student’s t-test). G, gilteritinib; ns, not statistically different.
