# Supplementary material for: A novel approach for relapsed/refractory FLT3mut+ acute myeloid leukaemia: synergistic effect of the combination of bispecific FLT3scFv/NKG2D-CAR T cells and gilteritinib
Source: Mol Cancer. 2022 Mar 4;21:66. doi: 10.1186/s12943-022-01541-9 (PMC8896098; doi:10.1186/s12943-022-01541-9)
Supplement: Supplementary file 3 — Additional file 3: Figure S3. FLT3 inhibitor Gilteritinib inhibits proliferation and induced apotosis in FLT3mut+AML cell lines. [file 12943_2022_1541_MOESM3_ESM.pptx]

## Slide 1
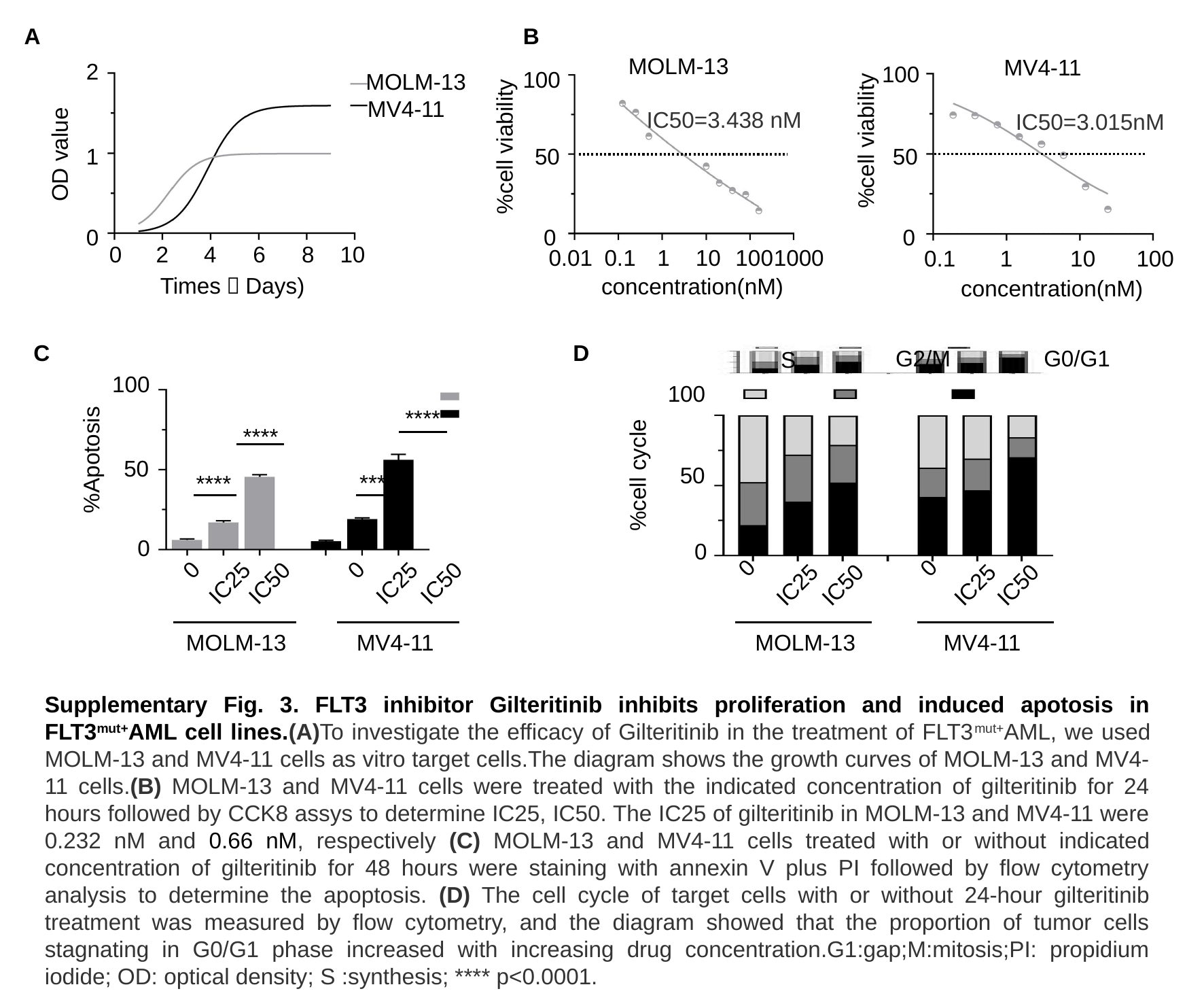

A
B
MOLM-13
100
IC50=3.438 nM
%cell viability
50
0
0.01
0.1
1
10
100
1000
concentration(nM)
MV4-11
100
IC50=3.015nM
%cell viability
50
0
0.1
1
10
100
concentration(nM)
2
MOLM-13
MV4-11
OD value
1
0
0
2
4
6
8
10
Times（Days)
C
D
G2/M
G0/G1
S
100
%cell cycle
50
0
0
0
IC25
IC50
IC25
IC50
MOLM-13
MV4-11
100
****
%Apotosis
****
50
****
****
0
0
0
IC25
IC50
IC25
IC50
MOLM-13
MV4-11
Supplementary Fig. 3. FLT3 inhibitor Gilteritinib inhibits proliferation and induced apotosis in FLT3mut+AML cell lines.(A)To investigate the efficacy of Gilteritinib in the treatment of FLT3mut+AML, we used MOLM-13 and MV4-11 cells as vitro target cells.The diagram shows the growth curves of MOLM-13 and MV4-11 cells.(B) MOLM-13 and MV4-11 cells were treated with the indicated concentration of gilteritinib for 24 hours followed by CCK8 assys to determine IC25, IC50. The IC25 of gilteritinib in MOLM-13 and MV4-11 were 0.232 nM and 0.66 nM, respectively (C) MOLM-13 and MV4-11 cells treated with or without indicated concentration of gilteritinib for 48 hours were staining with annexin V plus PI followed by flow cytometry analysis to determine the apoptosis. (D) The cell cycle of target cells with or without 24-hour gilteritinib treatment was measured by flow cytometry, and the diagram showed that the proportion of tumor cells stagnating in G0/G1 phase increased with increasing drug concentration.G1:gap;M:mitosis;PI: propidium iodide; OD: optical density; S :synthesis; **** p<0.0001.
