# Supplementary material for: A novel approach for relapsed/refractory FLT3mut+ acute myeloid leukaemia: synergistic effect of the combination of bispecific FLT3scFv/NKG2D-CAR T cells and gilteritinib
Source: Mol Cancer. 2022 Mar 4;21:66. doi: 10.1186/s12943-022-01541-9 (PMC8896098; doi:10.1186/s12943-022-01541-9)
Supplement: Supplementary file 4 — Additional file 4: Figure S4. FLT3scFv/NKG2D-CAR T cells show certain cytotoxicity aginst FLT3mut- AML cell lines in vitro [file 12943_2022_1541_MOESM4_ESM.pptx]

## Slide 1
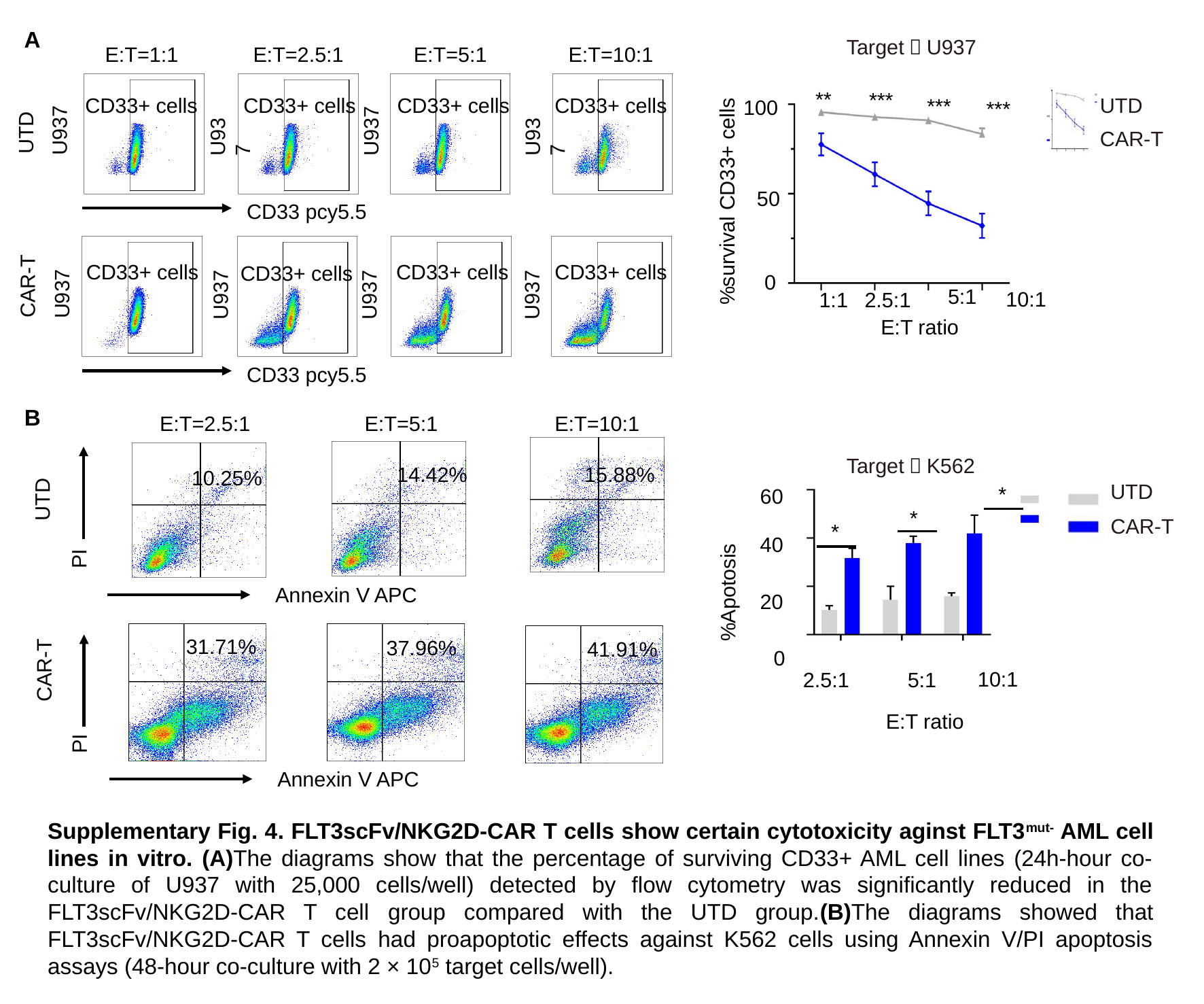

A
Target：U937
**
***
UTD
***
100
***
CAR-T
%survival CD33+ cells
50
0
5:1
10:1
 2.5:1
1:1
E:T ratio
E:T=1:1
E:T=2.5:1
E:T=5:1
E:T=10:1
U937
CD33+ cells
CD33+ cells
CD33+ cells
CD33+ cells
U937
U937
U937
UTD
CD33 pcy5.5
CAR-T
U937
CD33+ cells
CD33+ cells
CD33+ cells
U937
CD33+ cells
U937
U937
CD33 pcy5.5
B
E:T=2.5:1
E:T=5:1
E:T=10:1
15.88%
14.42%
10.25%
UTD
PI
Annexin V APC
31.71%
37.96%
41.91%
CAR-T
PI
Annexin V APC
Target：K562
UTD
*
60
*
CAR-T
*
40
%Apotosis
20
0
10:1
 2.5:1
5:1
E:T ratio
Supplementary Fig. 4. FLT3scFv/NKG2D-CAR T cells show certain cytotoxicity aginst FLT3mut- AML cell lines in vitro. (A)The diagrams show that the percentage of surviving CD33+ AML cell lines (24h-hour co-culture of U937 with 25,000 cells/well) detected by flow cytometry was significantly reduced in the FLT3scFv/NKG2D-CAR T cell group compared with the UTD group.(B)The diagrams showed that FLT3scFv/NKG2D-CAR T cells had proapoptotic effects against K562 cells using Annexin V/PI apoptosis assays (48-hour co-culture with 2 × 10^5^ target cells/well).
