# Supplementary material for: A novel approach for relapsed/refractory FLT3mut+ acute myeloid leukaemia: synergistic effect of the combination of bispecific FLT3scFv/NKG2D-CAR T cells and gilteritinib
Source: Mol Cancer. 2022 Mar 4;21:66. doi: 10.1186/s12943-022-01541-9 (PMC8896098; doi:10.1186/s12943-022-01541-9)
Supplement: Supplementary file 5 — Additional file 5: Figure S5. The transcriptional role of NF-κB2 in NKG2DL expression [file 12943_2022_1541_MOESM5_ESM.pptx]

## Slide 1
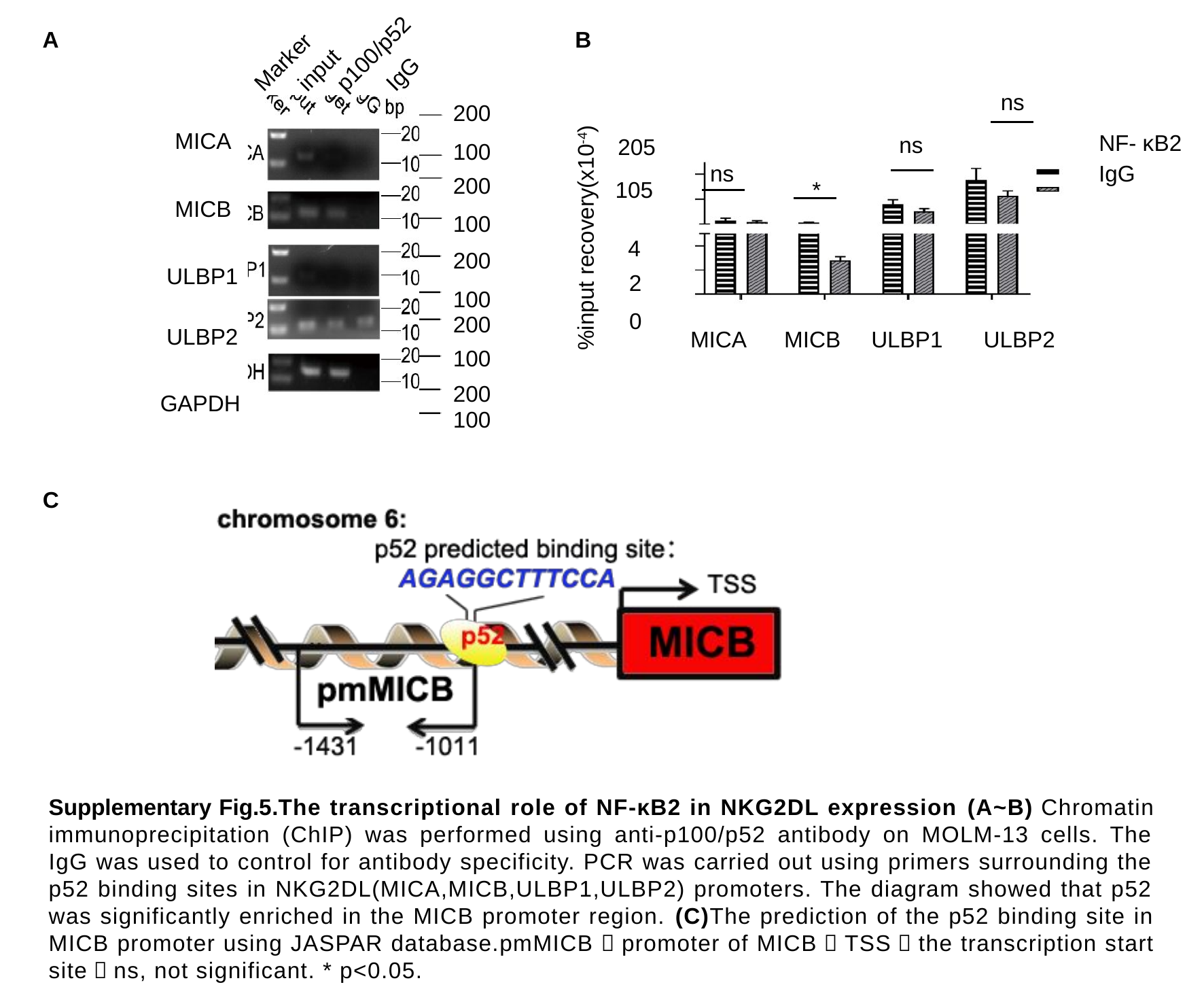

A
p100/p52
Marker
input
IgG
200
MICA
100
200
MICB
100
200
ULBP1
100
200
ULBP2
100
200
GAPDH
100
B
ns
NF- κB2
ns
205
ns
IgG
105
*
%input recovery(x10-4)
4
2
0
MICA
MICB
ULBP1
ULBP2
C
Supplementary Fig.5.The transcriptional role of NF-κB2 in NKG2DL expression (A~B) Chromatin immunoprecipitation (ChIP) was performed using anti-p100/p52 antibody on MOLM-13 cells. The IgG was used to control for antibody specificity. PCR was carried out using primers surrounding the p52 binding sites in NKG2DL(MICA,MICB,ULBP1,ULBP2) promoters. The diagram showed that p52 was significantly enriched in the MICB promoter region. (C)The prediction of the p52 binding site in MICB promoter using JASPAR database.pmMICB：promoter of MICB；TSS：the transcription start site；ns, not significant. * p<0.05.
