# Supplementary material for: A novel approach for relapsed/refractory FLT3mut+ acute myeloid leukaemia: synergistic effect of the combination of bispecific FLT3scFv/NKG2D-CAR T cells and gilteritinib
Source: Mol Cancer. 2022 Mar 4;21:66. doi: 10.1186/s12943-022-01541-9 (PMC8896098; doi:10.1186/s12943-022-01541-9)
Supplement: Supplementary file 6 — Additional file 6: Figure S6. Gilteritinib pretreatment enhanced the proliferation of FLT3scFv/NKG2D CAR-T cells [file 12943_2022_1541_MOESM6_ESM.pptx]

## Slide 1
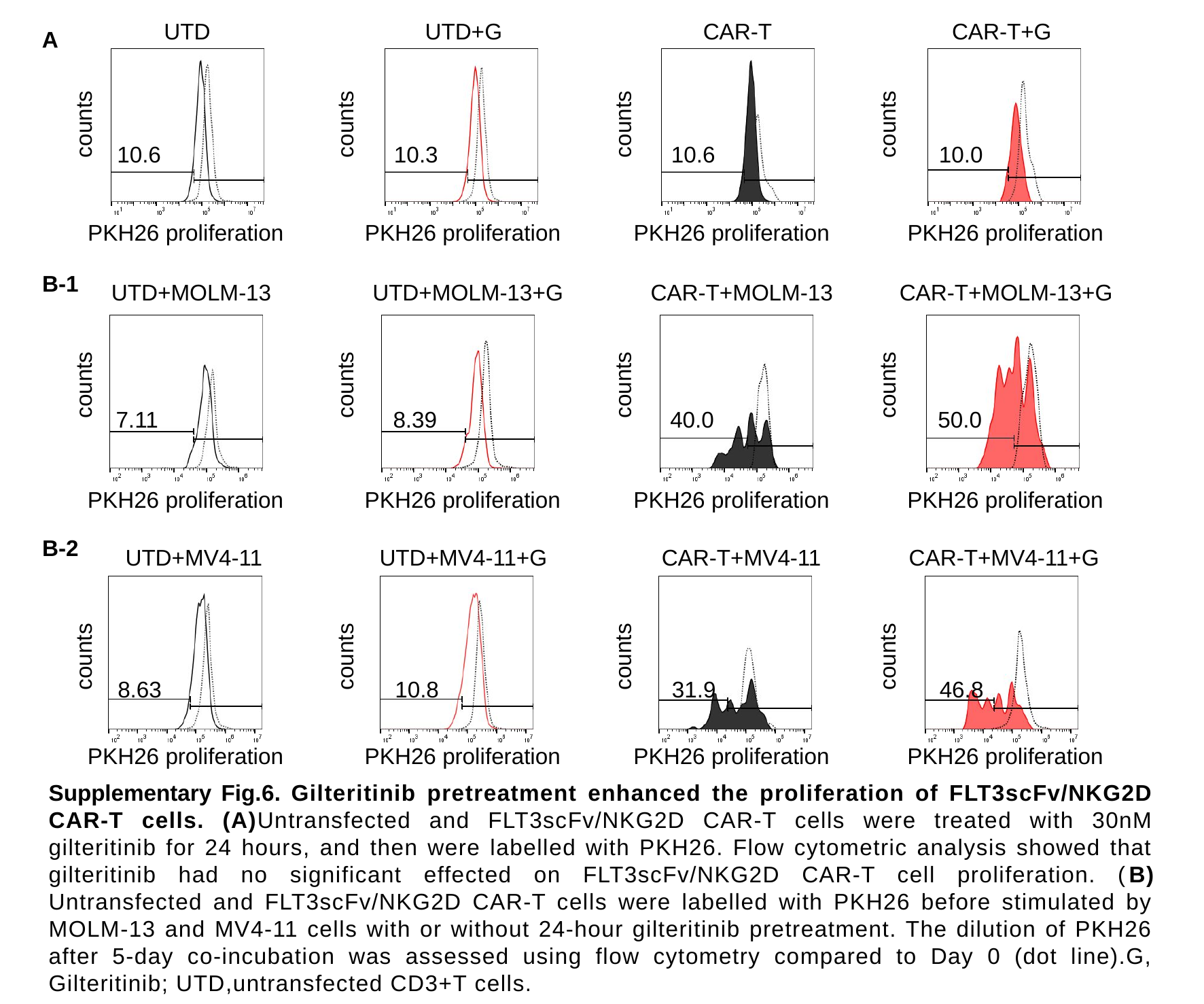

UTD
UTD+G
CAR-T
CAR-T+G
A
counts
counts
counts
counts
10.6
10.3
10.6
10.0
PKH26 proliferation
PKH26 proliferation
PKH26 proliferation
PKH26 proliferation
B-1
UTD+MOLM-13
UTD+MOLM-13+G
CAR-T+MOLM-13
CAR-T+MOLM-13+G
counts
counts
counts
counts
7.11
8.39
40.0
50.0
PKH26 proliferation
PKH26 proliferation
PKH26 proliferation
PKH26 proliferation
B-2
UTD+MV4-11
UTD+MV4-11+G
CAR-T+MV4-11
CAR-T+MV4-11+G
counts
counts
counts
counts
8.63
10.8
31.9
46.8
PKH26 proliferation
PKH26 proliferation
PKH26 proliferation
PKH26 proliferation
Supplementary Fig.6. Gilteritinib pretreatment enhanced the proliferation of FLT3scFv/NKG2D CAR-T cells. (A)Untransfected and FLT3scFv/NKG2D CAR-T cells were treated with 30nM gilteritinib for 24 hours, and then were labelled with PKH26. Flow cytometric analysis showed that gilteritinib had no significant effected on FLT3scFv/NKG2D CAR-T cell proliferation. (B) Untransfected and FLT3scFv/NKG2D CAR-T cells were labelled with PKH26 before stimulated by MOLM-13 and MV4-11 cells with or without 24-hour gilteritinib pretreatment. The dilution of PKH26 after 5-day co-incubation was assessed using flow cytometry compared to Day 0 (dot line).G, Gilteritinib; UTD,untransfected CD3+T cells.
