# Supplementary material for: A novel approach for relapsed/refractory FLT3mut+ acute myeloid leukaemia: synergistic effect of the combination of bispecific FLT3scFv/NKG2D-CAR T cells and gilteritinib
Source: Mol Cancer. 2022 Mar 4;21:66. doi: 10.1186/s12943-022-01541-9 (PMC8896098; doi:10.1186/s12943-022-01541-9)
Supplement: Supplementary file 7 — Additional file 7: Figure S7. Gilteritinib pretreatment promotes FLT3scFv/NKG2D CAR-T cell-target cell conjunction formation [file 12943_2022_1541_MOESM7_ESM.pptx]

## Slide 1
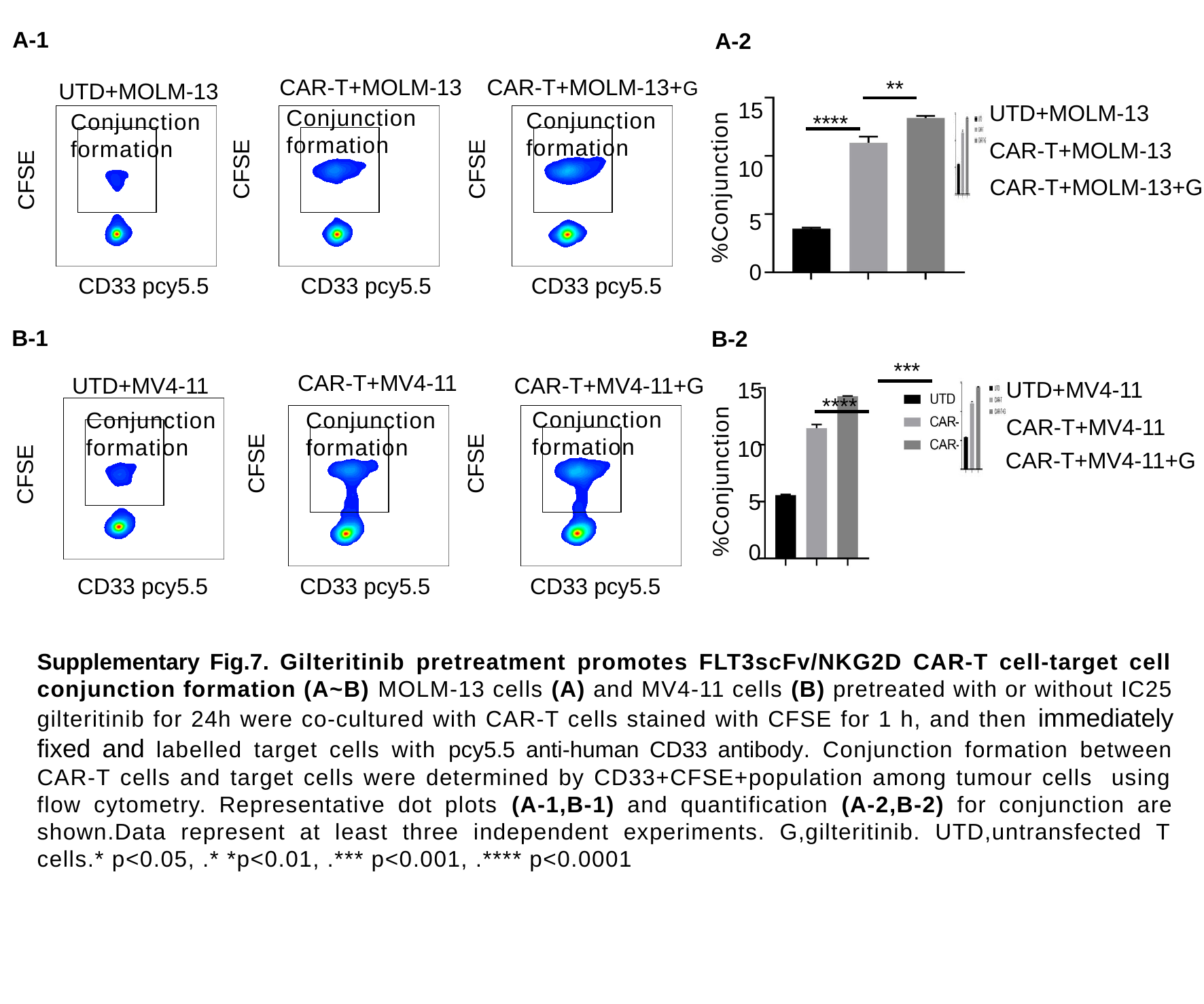

A-1
A-2
CAR-T+MOLM-13+G
CAR-T+MOLM-13
**
UTD+MOLM-13
15
UTD+MOLM-13
Conjunction formation
Conjunction formation
Conjunction formation
****
CAR-T+MOLM-13
CFSE
CFSE
10
CFSE
%Conjunction
CAR-T+MOLM-13+G
5
0
CD33 pcy5.5
CD33 pcy5.5
CD33 pcy5.5
B-1
B-2
***
CAR-T+MV4-11
UTD+MV4-11
CAR-T+MV4-11+G
UTD+MV4-11
15
****
Conjunction formation
Conjunction formation
Conjunction formation
CAR-T+MV4-11
10
CFSE
CFSE
CAR-T+MV4-11+G
CFSE
%Conjunction
5
0
CD33 pcy5.5
CD33 pcy5.5
CD33 pcy5.5
Supplementary Fig.7. Gilteritinib pretreatment promotes FLT3scFv/NKG2D CAR-T cell-target cell conjunction formation (A~B) MOLM-13 cells (A) and MV4-11 cells (B) pretreated with or without IC25 gilteritinib for 24h were co-cultured with CAR-T cells stained with CFSE for 1 h, and then immediately fixed and labelled target cells with pcy5.5 anti-human CD33 antibody. Conjunction formation between CAR-T cells and target cells were determined by CD33+CFSE+population among tumour cells using flow cytometry. Representative dot plots (A-1,B-1) and quantification (A-2,B-2) for conjunction are shown.Data represent at least three independent experiments. G,gilteritinib. UTD,untransfected T cells.* p<0.05, .* *p<0.01, .*** p<0.001, .**** p<0.0001
