# Supplementary material for: A novel approach for relapsed/refractory FLT3mut+ acute myeloid leukaemia: synergistic effect of the combination of bispecific FLT3scFv/NKG2D-CAR T cells and gilteritinib
Source: Mol Cancer. 2022 Mar 4;21:66. doi: 10.1186/s12943-022-01541-9 (PMC8896098; doi:10.1186/s12943-022-01541-9)
Supplement: Supplementary file 8 — Additional file 8: Figure S8. The properties of FLT3scFv/NKG2D CAR T cells as compared to UTD cells. [file 12943_2022_1541_MOESM8_ESM.pptx]

## Slide 1
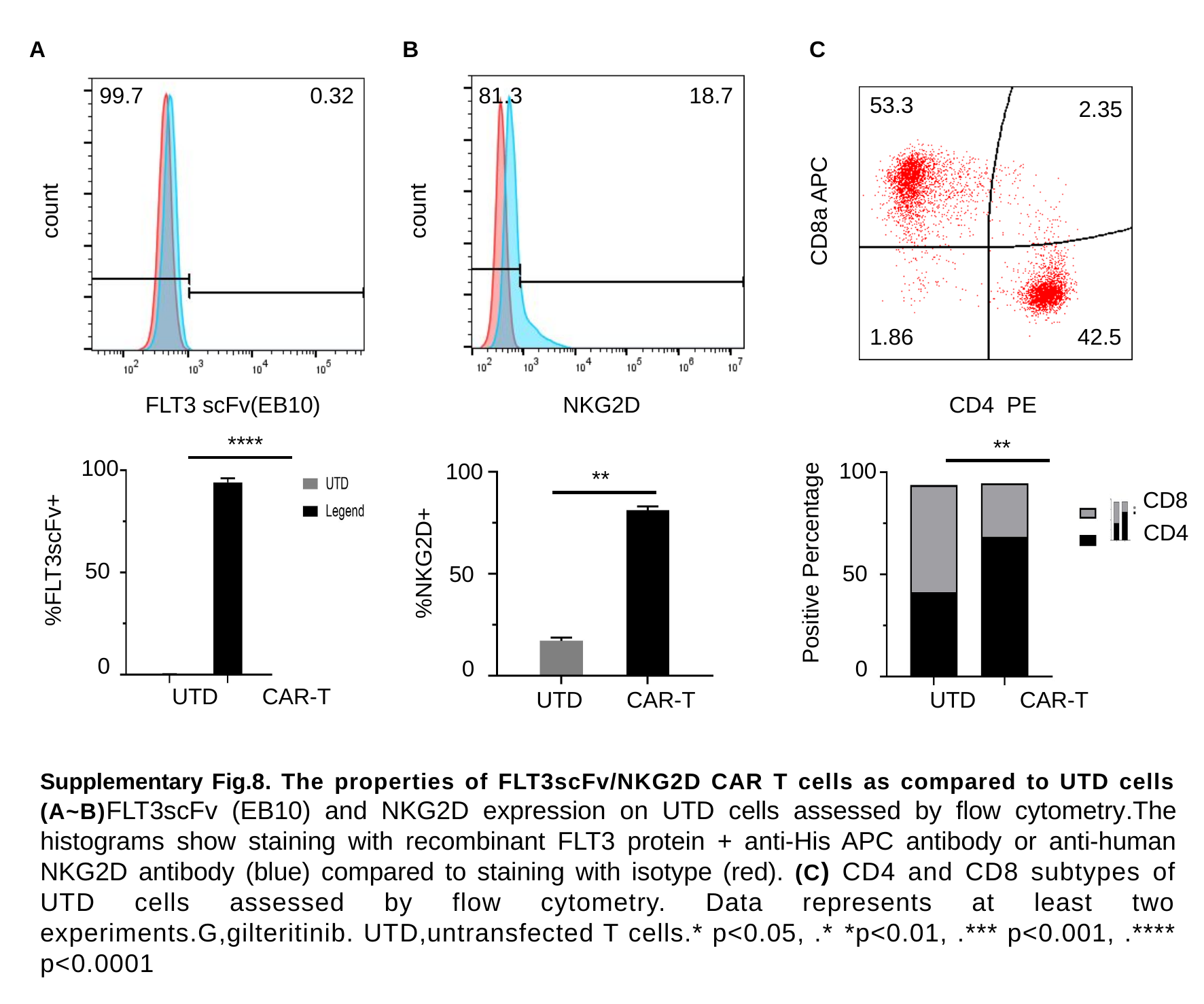

A
B
C
99.7
0.32
81.3
18.7
53.3
2.35
CD8a APC
count
count
1.86
42.5
FLT3 scFv(EB10)
NKG2D
CD4 PE
****
**
100
100
100
**
Positive Percentage
CD8
%FLT3scFv+
%NKG2D+
CD4
50
50
50
0
0
0
UTD
CAR-T
UTD
CAR-T
UTD
CAR-T
Supplementary Fig.8. The properties of FLT3scFv/NKG2D CAR T cells as compared to UTD cells (A~B)FLT3scFv (EB10) and NKG2D expression on UTD cells assessed by flow cytometry.The histograms show staining with recombinant FLT3 protein + anti-His APC antibody or anti-human NKG2D antibody (blue) compared to staining with isotype (red). (C) CD4 and CD8 subtypes of UTD cells assessed by flow cytometry. Data represents at least two experiments.G,gilteritinib. UTD,untransfected T cells.* p<0.05, .* *p<0.01, .*** p<0.001, .**** p<0.0001
