# Supplementary material for: A novel approach for relapsed/refractory FLT3mut+ acute myeloid leukaemia: synergistic effect of the combination of bispecific FLT3scFv/NKG2D-CAR T cells and gilteritinib
Source: Mol Cancer. 2022 Mar 4;21:66. doi: 10.1186/s12943-022-01541-9 (PMC8896098; doi:10.1186/s12943-022-01541-9)
Supplement: Supplementary file 9 — Additional file 9: Figure S9. Cytokine secretion of UTD cells and FLT3scFv/NKG2D CAR-T after coculture with target cells. [file 12943_2022_1541_MOESM9_ESM.pptx]

## Slide 1
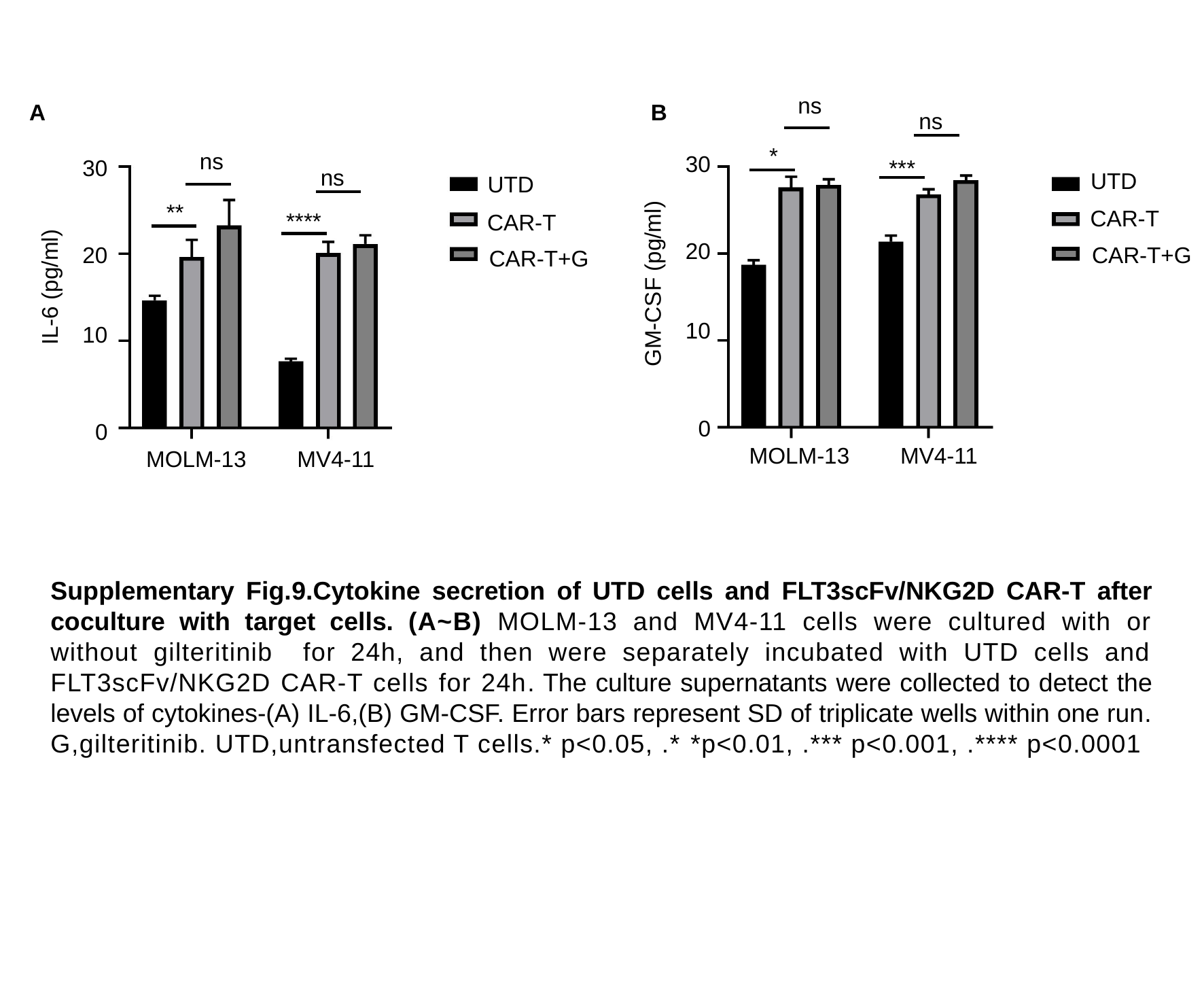

ns
A
B
ns
*
ns
30
30
***
ns
UTD
UTD
**
GM-CSF (pg/ml)
CAR-T
****
CAR-T
IL-6 (pg/ml)
20
20
CAR-T+G
CAR-T+G
10
10
0
0
MOLM-13
MV4-11
MOLM-13
MV4-11
Supplementary Fig.9.Cytokine secretion of UTD cells and FLT3scFv/NKG2D CAR-T after coculture with target cells. (A~B) MOLM-13 and MV4-11 cells were cultured with or without gilteritinib for 24h, and then were separately incubated with UTD cells and FLT3scFv/NKG2D CAR-T cells for 24h. The culture supernatants were collected to detect the levels of cytokines-(A) IL-6,(B) GM-CSF. Error bars represent SD of triplicate wells within one run. G,gilteritinib. UTD,untransfected T cells.* p<0.05, .* *p<0.01, .*** p<0.001, .**** p<0.0001
