# Supplementary material for: A novel approach for relapsed/refractory FLT3mut+ acute myeloid leukaemia: synergistic effect of the combination of bispecific FLT3scFv/NKG2D-CAR T cells and gilteritinib
Source: Mol Cancer. 2022 Mar 4;21:66. doi: 10.1186/s12943-022-01541-9 (PMC8896098; doi:10.1186/s12943-022-01541-9)
Supplement: Supplementary file 10 — Additional file 10: Supplementary methods [file 12943_2022_1541_MOESM10_ESM.docx]

**Supplementary methods**

**METHODS**

**Lentivirus production**

The day before transfection, 293T cells were seeded in a 10 cm petri dish and cultured overnight in high-glucose DMEM (Gibco, USA) supplemented with 10% foetal bovine serum (FBS; Gibco, USA). Once they reached 60%~80% confluency, the cells were mixed with the transfection complex including 30 μL Lenti-EF1a-FLT3scFv-CAR-NKG2D-CAR-EGFP plasmid, 10 μg Lenti-GOI and 50 μL LVTransm (all from iCARTAB, China). After 6 to 8 hours, the medium containing the transfection reagent was replaced with fresh high-glucose DMEM supplemented with 10% FBS. The supernatant containing virus was harvested at 24 hours and 48 hours, centrifuged at 4°C and 50,000 x g for 120 minutes to pellet virus particles, and then stored at -80°C for CAR T cell generation.

**T cell isolation and transduction**

Peripheral blood mononuclear cells (PBMCs) were separated from the peripheral blood of three healthy donors using Ficoll-Paque density gradient medium (TBD Science, China). The cells were enriched using Pan T Cell Biotin-Antibody Cocktail and Pan T Cell MicroBead Cocktail (Miltenyi, Germany) in accordance with the manufacturer’s instructions. Finally, CD3+ T cells were obtained with the use of an MS sorting column (Miltenyi, Germany), seeded at 1 x 10^6 cells/ml in TexMACS GMP medium (Miltenyi, Germany) containing 200 IU/ml IL-2, 10 ng/ml IL-7 and 5 ng/ml IL-15 at 37°C in 5% CO2, and then activated using 25 µl/ml ImmunoCult™ Human CD3/CD28 T Cell Activator (StemCell, Canada). After 24 hours, lentiviral particles were added to the cultures at a multiplicity of infection (MOI) of 10 in the presence of polybrene (Sigma, USA) at a final concentration of 10 µg/ml. The CAR T cells were counted on alternate days and suspended in fresh medium to maintain the cell density at 1 × 10^6^ cells/ml. Between approximately 7 and 14 days after infection, CAR T cells were collected for in vitro and in vivo experiments.

**ELISA**

Gilteritinib treated and untreated AML cells (5 × 10^5^ cells per well in 1ml medium) were incubated with CAR-T cells at the E:T ratio of 5:1 in 24-well plates for 24h. The supernatant was collected to detect the expression of IL-2 and IFN-γ according to the instructions of ELISA kit (Dakewe, China) and the absorbance at 450 nm was measured using Synergy H4 Hybrid Microplate Reader (Thermo Scientific,USA).

**Cytotoxicity Assay**

In order to verify the specific cytotoxicity of CAR-T cells against AML cells, target cells were co-cultured with CAR-T cells at the E:T ratios of 5:1(with 10,000 target cells/well),10:1,20:1 and 40:1in 96-well V-bottom plates. After incubation at 37℃ for 4 hours, 50µl of supernatant were subjected for CytoTox96 non-radioactive cytotoxicity assay (Promega, USA) according to the manufacturer’s instruction. The absorbance was recorded at 490nm within 1 hour after the addition of stop solution. Cytotoxicity (%) = (Experimental-Effector spontaneous-Target spontaneous) *100/(Target maximum-Target spontaneous).

**Apoptotic analysis**

Apoptosis of AML cells was determined using Annexin V-APC Apoptosis Detection kit (KeyGEN). Target cells were incubated with various concentrations of CAR-T cells for 48h in 24-well plates at a density of 2 × 10^5^ cells per well. The cells were washed with cold PBS twice and then re-suspended with 500μl 1× binding buffer. 5μl of Annexin V-APC (KeyGEN) and 5μl of propidium iodide (KeyGEN) was added to each well before being left to incubate in the dark for 25 min. The fluorescent intensities were determined using flow cytometry (Becton-Dickinson-San Jose CA, USA). A minimum of 30,000 cells were collected from each sample.

**Cell survival evaluation**

The target cells (25,000 cells/well) in 1ml medium (containing 5 %FBS TexMACS Medium) were co-cultured in 24-well plates with PBS or FLT3scFv/NKG2D-CAR T cells at E:T ratios of 1:1, 2.5:1, 5:1, 10:1 for 24 hours. The cells were collected, washed once with PBS, stained with anti-CD33-Percp-Cy5.5 (Biolegend, USA) at 4°C for 30 min, and then washed and re-suspended in PBS for flow cytometry analysis. The percentage of CD33^+^ cells represents the survival level of target cells.

**Immunofluorescence staining**

Mouse femurs were harvested and fixed in 10% neutral buffered formalin for one day and then paraffin-embeded and sectioned (20μm thickness). The slides were incubated with 1% BSA, 22.52 mg/mL glycine in PBST (PBS + 0.1% Tween 20) for 30 minutes and then anti-GFP antibody (Proteintech, USA) (dissolved in 1% serum PBST) at room temperature for 1 hour. After being washed with PBS 3 times, slides were incubated for 1 hour in the dark with antibodies for genes to be examined. This was followed by counter-staining with 0.1-1 μg/mL DAPI for 1 minute, rinsed with PBS, and mounted. The distribution of CAR-T cells and the cells expressing ULBP1 and FLT3 were detected by fluorescence microscopy (Nikon, USA).

**Real-time PCR**

Cells were lysed with Trizol (TIANGEN, China) to extract total RNA.Then cDNA was synthesized according to the manufacturer’s instruction. The reaction condition established for real-time PCR was: 95℃ for 10 minutes, and 40 cycles of 55℃ for 20 seconds each and 72℃ for 35 seconds. Gene expressions relative to GAPDH were calculated by 2-ΔΔCt method. The forward/reverse primer sequences were listed in Supplementary Table 4.

**Western blotting**

Cells lysates were prepared for Western blotting analysis according to standardized protocol. In brief, total proteins were separated by SDS-PAGE and transferred to polyvinylidene fluoridemembrane (Millipore, USA). After blocking with 5% skim milk at 37℃ for 2 hours, the membranes were incubated with primary antibodies (1:500) at 4℃ for 12 hours and secondary antibody HRP-Goat Anti-Mouse IgG (H+L) (Proteintech, USA) (1:10000) at 37℃ for 1 hour, respectively. Primary antibodies included antibodies against FLT3 (Proteintech，USA), MICA(R&D SYSTEMS，USA), MICB (R&D SYSTEMS，USA), ULBP1 (Abcam, USA),ULBP2 (R&D SYSTEMS，USA), p65，p-p65，p100/p52, p-p100 (Cell Signaling Technology, USA). GAPDH and β-actin (Proteintech, USA) were used as internal controls. The gray intensity of protein bands were semi-quantified using Image J software. All antibodies were listed in Supplementary table 3.

**Chromatin immunoprecipitation (ChIP) assay**

In vitro binding of NF-κB subunits to the target sites in the NKG2DLs promoters were tested with ChIP assay using NF-κB2 /p100 antibody and the Pierce^TM^Agarose ChIP kit (Thermo, USA) according to the manufacturer's instructions. Briefly, cells were cross-linked with 1% formaldehyde and then pre-cleared with Protein A/G PLUS Agarose (Biyuntian, China). For the immunoprecipitation assay, cell lysates were incubated with antibodies against NF-κB2 (Cell Signaling Technology) at 4°C overnight. Rabbit IgG was used as a negative control, whereas anti-RNA polymerase II antibody (#1862243; Thermo Fisher Scientific, Inc.) was employed as a positive control. As loading controls, 10% total input samples were used. Promoters were determined using qPCR. All gene sequences are shown in Supplementary Table 5.

**Luciferase assay**

Genomic DNA fragment of MICB promoter was generated by PCR and inserted into pGL3-Basic vectors (denoted as pGL3–MICB) (Promega, USA) using T4 DNA Ligase (Promega, USA). After introducing pGL3-MICB into TOP10 competent bacteria, positive monoclones were selected to extract the plasmid, which was confirmed by DNA sequencing. Some MOLM-13 cells (6×10^4^ cells/well) were transiently transfected with 50nM si-RNA(siRNA-NC or siRNA- NF-κB2) for 72 hours.24 hours after tranfection,MOLM-13 cells (6×10^4^ cells/well) were transfected with 0.5 μg of plasmid and 0.5ml of opti-MEM (Gibco, USA) using Lipofectamine 3000 (Invitrogen, USA) in 6-well plates MOLM-13 cells.The plasmids contain pGL3.1 constructs with or witout MICB promoter and a pRL-TK vector (Promega). pRL-TK vector was used to normalize for luciferase activity.24 hours later, the cells were cultured with or without gilteritinib treatment for 24 hours. Subsequently, the cells were prepared for luciferase assays using a dual luciferase assay system (Promega) following the manufacturer’s protocol. Luciferase activity was determined by relative light unit (RLU) using Dual Luciferase Assay System (Promega, USA).

**Toxicity of FLT3scFv/NKG2D-CAR T cells to normal hematopoietic stem cells (HSCs)**

According to the manufacturer's instructions, CD34+ cells were separated from neonatal cord blood after labeling with CD34 magnetic beads (Miltenyi Biotec, Germany). The isolated CD34 cells were added with 5µl anti-CD34-APC (Biolegend, USA) to determine the viability and purity of the sorted CD34+ cells by flow cytometry. The expression of NKG2DLs and FLT3 on CD34+ cells were determined using flow cytometry.

CD34+ cells were treated with un-transduced CD3+T cells (UTD), CAR T cells, Gilteritinib + CAR T cells respectively for 4 hours and then labelled with 5ul anti-CD34 APC and 50ul of 7-ADD dye solution (KeyGEN, China) according to manufacturer’s instructions. Cytotoxicity was determined using flow cytometry.

500 CD34+ cells co-cultured with CAR T cells or UTD cells in each group were inoculated into methylcellulose medium (H4434, Steamcell, Cananda) and incubated with 5% CO2 at 37°C. After 14 days, the GEMM (granulocyte/erythroid/macrophage/megakaryocyte, 500 cells/colony), CFU-G/M (granulocyte–macrophage progenitor, 40 cells/colony) colony and BFU-E (erythroid progenitors, 200 cells/colony) were counted under Nikon Eclipse Ti-U fluorescence microscope (Nikon, Japan)
