# Supplementary material for: A novel approach for relapsed/refractory FLT3mut+ acute myeloid leukaemia: synergistic effect of the combination of bispecific FLT3scFv/NKG2D-CAR T cells and gilteritinib
Source: Mol Cancer. 2022 Mar 4;21:66. doi: 10.1186/s12943-022-01541-9 (PMC8896098; doi:10.1186/s12943-022-01541-9)
Supplement: Supplementary file 11 — Additional file 11: Table S1. The sequences of FLT3scFv and NKG2D utilised in CAR [file 12943_2022_1541_MOESM11_ESM.docx]

**Supplementary Table 1. The sequence of FLT3scFv and NKG2D utilised in CAR**

|  | sequence | antibody |
| --- | --- | --- |
| NKG2D extracellular domain sequence | ATATGGAGTGCCGTATTCCTAAATTCATTATTCAACCAAGAAGTTCAAATTCCCTTGACTGAAAGTTACTGTGGCCCATGTCCTAAAAACTGGATATGTTATAAAAATAACTGCTACCAGTTTTTTAATGAGAGTAAAAACTGGTATGAGAGCCAGGCTTCTTGTATGTCTCAAAATGCCAGCCTTCTGAAAGTATACAGCAAAGAGGACCAGGATTTACTTAAACTGGTGAAGTCATATCACTGGATGGGATTAGTACACATTCCAACAAATGGATCTTGGCAGTGGGAAGATGGCTCCATTCTCTCACCCAACCTACTAACAATAATTGAAATGCAGAAGGGAGACTGTGCACTCTATGCCTCAAGCTTTAAAGGCTACATAGAAAACTGTTCGATTCCAAATACATACATCTGCATGCAGAGGACTGTG | - |
| FLT3scFv | GATGTTGTGATGACTCAGTCTCCACTCTCCCTGCCCGTCACCCCTGGAGAGCCGGCCTCCATCTCCTGCAGGTCTAGTCAGAGCCTCCTGCATAGTAATGGAAACAACTATTTGGATTGGTACCTGCAGAAGCCAGGGCAGTCTCCACAGCTCCTGATCTATTTGGGTTCTAATCGGGCCTCTGGGGTCCCAGACAGATTCAGCGGCAGTGGGTCAGACACTGATTTCACACTGCAAATCAGTAGGGTGGAGGCTGAGGATGTTGGGGTTTATTACTGCATGCAAGGTACACACCCCGCCATCTCCTTCGGCCAAGGGACACGACTGGAGATTAAAGGTGGCGGAGGATCTGGCGGAGGTGGAAGCGGCGGAGGCGGATCTGAGGTCCAGCTGGTGCAGTCTGGGGCTGAGGTGAAGAAGCCTGGGGCCTCAGTGAAGGTTTCCTGCAAGGCATCTGGATACACCTTCACCAGCTACTATATGCACTGGGTGCGACAGGCCCCTGGACAAGGGCTTGAGTGGATGGGAATAATCAACCCTAGTGGTGGTAGCACAAGCTACGCACAGAAGTTCCAGGGCAGAGTCACCATGACCAGGGACACGTCCACGAGCACAGTCTACATGGAGCTGAGCAGCCTGAGATCTGAGGACACGGCCGTGTATTACTGTGCGAGGGGAGTGGGAGCGCATGATGCTTTTGATATCTGGGGCCAAGGGACCACGGTCACCGTCTCAAGC | EB10 |
