# Supplementary material for: A novel approach for relapsed/refractory FLT3mut+ acute myeloid leukaemia: synergistic effect of the combination of bispecific FLT3scFv/NKG2D-CAR T cells and gilteritinib
Source: Mol Cancer. 2022 Mar 4;21:66. doi: 10.1186/s12943-022-01541-9 (PMC8896098; doi:10.1186/s12943-022-01541-9)
Supplement: Supplementary file 12 — Additional file 12: Table S2. Clinical characteristics of patients with acute myeloid leukaemia (AML) [file 12943_2022_1541_MOESM12_ESM.docx]

**Supplementary Table 2. Clinical characteristics of patients with acute myeloid leukaemia (AML)**

| **Patient** | **Age**  **(years)** | **Sex** | **Diagnosis** | **Cytogenetics** | **FLT3 classification** | **CD33+ cell %** |
| --- | --- | --- | --- | --- | --- | --- |
| 1 | 34 | Male | AML-M5 | WT1+ | FLT3^mut-^ AML | 89.6 |
| 2 | 23 | Female | AML-M5 | WT1+; CEBPA+ | FLT3^mut-^ AML | 80.5 |
| 3 | 67 | Female | AML-M5 | CEBPA+ | FLT3^mut-^ AML | 95.5 |
| 4 | 57 | Male | AML-M5 | IDH2+ | FLT3^mut-^ AML | 85.4 |
| 5 | 29 | Male | AML-M5 | WT1+ | FLT3^mut-^ AML | 90.25 |
| 6 | 21 | Female | AML-M5 | FLT3-ITD high; WT1+ | FLT3^mut+^ AML | 83.7 |
| 7 | 60 | Female | AML-M5 | FLT3-ITD high; CEBPa+ | FLT3^mut+^ AML | 92.5 |
| 8 | 49 | Female | AML-M2 | FLT3-ITD high; WT1+; | FLT3^mut+^ AML | 88.0 |
| 9 | 59 | Male | AML-M5 | FLT3-ITD high, MLL-AF6/AF10/ELL/ENL+, CEBPA+, KRAS+ | FLT3^mut+^ AML | 90.0 |
| 10 | 61 | Male | AML-M5 | FLT3-ITD high | FLT3^mut+^ AML | 94.3 |

*WT1*:Wilm tumor gene1;*CEBPA*:CCAAT enhancer binding protein alpha; *IDH2*:isocitrate dehydrogenase (NADP(+)) 2; *FLT3-ITD*: FMS-like tyrosine kinase-3 internal tandem duplication;MLL-AF6/AF10/ELL/ENL+;*KRAS*:KRAS proto-oncogene, GTPase；AML-M5: Acute myeloid leukemia-Monocytic leukemia
