# Supplementary material for: A novel approach for relapsed/refractory FLT3mut+ acute myeloid leukaemia: synergistic effect of the combination of bispecific FLT3scFv/NKG2D-CAR T cells and gilteritinib
Source: Mol Cancer. 2022 Mar 4;21:66. doi: 10.1186/s12943-022-01541-9 (PMC8896098; doi:10.1186/s12943-022-01541-9)
Supplement: Supplementary file 13 — Additional file 13: Table S3. Antibodies used for flow cytometry and Western blot [file 12943_2022_1541_MOESM13_ESM.docx]

**Supplementary Table 3. Antibodies used for flow cytometry and Western blot**

| **Name of antibodies** | **Suppliers** |
| --- | --- |
| **Flow cytometry:** |  |
| APC anti-human CD135 (Flt-3/Flk-2) | Biolegend，USA |
| Alexa Fluor® 488 anti-human MICA/MICB | Biolegend，USA |
| Anti-human ULBP1 PE | Novus，USA |
| Alexa Fluor® 488 anti-human ULBP2/5/6 | R&D SYSTEMS，USA |
| Alexa Fluor® 488 anti-human ULBP3 | R&D SYSTEMS，USA |
| Anti-Human CD314 (NKG2D) PE | Biolegend，USA |
| APC anti-human CD34 | Biolegend，USA |
| APC anti-His Tag | Biolegend，USA |
| PerCP/Cyanine5.5 anti-human CD33 | Biolegend，USA |
| **Western blot:** |  |
| FLT3 Rabbit Polyclonal Antibody | Proteintech，USA |
| MICA Mouse Monoclonal Antibody | R&D SYSTEMS，USA |
| MICB Mouse Monoclonal Antibody | R&D SYSTEMS，USA |
| ULBP1 Rabbit Monoclonal Antibody | Abcam，USA |
| ULBP2 Rabbit Monoclonal Antibody | R&D SYSTEMS，USA |
| HRP-Goat Anti-Rabbit IgG（Goat） | Proteintech，USA |
| HRP-Goat Anti-Mouse IgG（Goat） | Proteintech，USA |
| Phospho-NF-κB/p65 (Ser536) (93H1) Rabbit Monoclonal Antibody | Cell Signaling Technology，USA |
| NF-κB/p65 Rabbit Polyclonal Antibody | Proteintech，USA |
| Phospho-NF-κB2/p100 (Ser866/870) Rabbit Polyclonal Antibody | Cell Signaling Technology，USA |
| NF-κB2 p100/p52 Rabbit Polyclonal Antibody | Cell Signaling Technology，USA |
| GAPDH Mouse Monoclonal Antibody | Proteintech，USA |
| β-actin Rabbit Polyclonal Antibody | Proteintech，USA |
