# Supplementary material for: A novel approach for relapsed/refractory FLT3mut+ acute myeloid leukaemia: synergistic effect of the combination of bispecific FLT3scFv/NKG2D-CAR T cells and gilteritinib
Source: Mol Cancer. 2022 Mar 4;21:66. doi: 10.1186/s12943-022-01541-9 (PMC8896098; doi:10.1186/s12943-022-01541-9)
Supplement: Supplementary file 14 — Additional file 14: Table S4. The sequences of forward/reverse primers for target genes [file 12943_2022_1541_MOESM14_ESM.docx]

**Supplementary Table 4.** **The sequences of forward/reverse primers for target genes**

| Target gene | Reference sequence | Name | Sequence (5' → 3') | Nucleotide position |
| --- | --- | --- | --- | --- |
| h-bax | NM_001291430.1 | Forward | TCTGGAGCAGGTCACAGT | 91 |
|  |  | Reverse | GGGATTGATCAGACACGTAAGG |  |
| h-bid | NM_197966.2 | Forward | CCTTGCTCCGTGATGTCTTT | 99 |
|  |  | Reverse | CCGTTCAGTCCATCCCATTT |  |
| h-puma | NM_001127240.3 | Forward | GTGACCACTGGCATTCATTTG | 129 |
|  |  | Reverse | TCCTCCCTCTTCCGAGATTT |  |
| h-p53 | NM_000546.5 | Forward | GTACCACCATCCACTACAACTAC | 141 |
|  |  | Reverse | CACAAACACGCACCTCAAAG |  |
| h-bcl2 | NM_000633.2 | Forward | GGCCAGGGTCAGAGTTAAATAG | 98 |
|  |  | Reverse | GGAGGTTCTCAGATGTTCTTCTC |  |
| h-mcl1 | NM_021960.5 | Forward | GTGAAGATGGTAGGGTGGAAAG | 93 |
|  |  | Reverse | TCGGCGGGTAATCAATTCTATG |  |
| h-ap-1 | NM_002228.4 | Forward | CCTGATGTACCTGATGCTATGG | 96 |
|  |  | Reverse | CCTCCTGAAACATCGCACTAT |  |
| h-ATM | NM_000051.3 | Forward | GCTCAGGAAGGAATGAGAGAAA | 103 |
|  |  | Reverse | CCACAGCTATCAACGTCAGTAA |  |
| h-ATR | NM_001184.4 | Forward | CACTGCTGGTTTGAGACCTATT | 101 |
|  |  | Reverse | CTGCTGACTTTGGTAGCATACA |  |
| h-CHK1 | NM_001114121.2 | Forward | GTAGATATGAAGCGTGCCGTAG | 113 |
|  |  | Reverse | CCTTCTCTCCTGTGACCATAGA |  |
| h-CHK2 | NM_007194.4 | Forward | CCTTAGTGGGTATCCACCTTTC | 122 |
|  |  | Reverse | CCAGAGCTTTCTCTGAGACTTC |  |
| h-GSK3β | NM_002093.4 | Forward | CCTCTGGCTACCATCCTTATTC | 101 |
|  |  | Reverse | CGGTCTCCAGTATTAGCATCTG |  |
| h-NF-KB1 | NM_001165412.2 | Forward | CTCCACAAGGCAGCAAATAGA | 106 |
|  |  | Reverse | ACTGGTCAGAGACTCGGTAAA |  |
| h-NF-KB2 | NM_001077494.3 | Forward | GGACTGTCACTTGGTGATACAG | 132 |
|  |  | Reverse | TGTCTGTCGGTACGTGTCTA |  |
| h-RelA | NM_001243984.2 | Forward | TGGGAATCCAGTGTGTGAAG | 125 |
|  |  | Reverse | CACAGCATTCAGGTCGTAGT |  |
| h-RelB | NM_006509.4 | Forward | CAATCCCAACCAGGATGTCT | 84 |
|  |  | Reverse | TCTCGCCAAGGCTGAATATG |  |
| h-C-Rel | NM_001291746.2 | Forward | CAATTCTCTCAGGCCTCCATAC | 112 |
|  |  | Reverse | GCAGGACCAACCTTCATACA |  |
| GAPDH | NM_001256799.2 | Forward | GGAGTCAACGGATTTGGTCGT | 108 |
|  |  | Reverse | GCTTCCCGTTCTCAGCCTTGA |  |
| h-NKG2D | - | Forward | AAGTTACTGTGGCCCATGTC | 102 |
|  |  | Reverse | CAAGAAGCCTGGCTCTCATAC |  |
| h-FLT3 scFv | - | Forward | GGGACACGACTGGAGATTAAAG | 101 |
|  |  | Reverse | TGCAGGAAACCTTCACTGAG |  |
