# Supplementary material for: A novel approach for relapsed/refractory FLT3mut+ acute myeloid leukaemia: synergistic effect of the combination of bispecific FLT3scFv/NKG2D-CAR T cells and gilteritinib
Source: Mol Cancer. 2022 Mar 4;21:66. doi: 10.1186/s12943-022-01541-9 (PMC8896098; doi:10.1186/s12943-022-01541-9)
Supplement: Supplementary file 15 — Additional file 15: Table S5. The sequences of forward/reverse primers for chromatin immunoprecipitation (ChIP) assay [file 12943_2022_1541_MOESM15_ESM.docx]

**Supplementary Table 5. The sequences of forward/reverse primers for chromatin immunoprecipitation (ChIP) assay**

| Target gene | Location | Primer name | Sequence |
| --- | --- | --- | --- |
| MICA | 1 | Forward | gcctgtaatcccagcactttg |
|  |  | Reverse | tagctgggattacaggcattcac |
| MICA | 2 | Forward | agactgcttaggcagtggcat |
|  |  | Reverse | tctactctaccgtggaaaacctcag |
| MICA | 3 | Forward | tgtagtgaaccacagggagcc |
|  |  | Reverse | gtttcagctttataatctccttctcctc |
| MICB | 1 | Forward | tggctaggtttgaagaccactg |
|  |  | Reverse | ttaaaacgataaattatgtgtggcatg |
| MICB | 2 | Forward | caaaacgcttgcgggc |
|  |  | Reverse | gtatttttagtagaggcggggtttc |
| MICB | 3 | Forward | gagtccagggatctaaggcaag |
|  |  | Reverse | tctcagcgccgaggc |
| ULBP1 | 1 | Forward | agcctccttctgccttcctc |
|  |  | Reverse | gtgagaaaagcctcccatgtaca |
| ULBP1 | 2 | Forward | gagaggcaaaaaaggttcgct |
|  |  | Reverse | atactcagtggcggcggt |
| ULBP1 | 3 | Forward | gagagttgcgtcagccagg |
|  |  | Reverse | tcacaccacggctgtttataaag |
| ULBP2 | 1 | Forward | agctttgaagggtggctgg |
|  |  | Reverse | cagttcagcagcctccctc |
| ULBP2 | 2 | Forward | aacctctggaaccctgcct |
|  |  | Reverse | ttgttggccaggctggt |
| ULBP2 | 3 | Forward | gttgtgatgattaaatgagtctgtcg |
|  |  | Reverse | aatatgattggctgccctgac |
| GAPDH | 1 | Forward | CATGGGTGTGAACCATGAGA |
|  |  | Reverse | GTCTTCTGGGTGGCAGTGAT |

MIC: MHC class I polypeptide-related sequence;ULBP: UL16 binding protein GAPDH: glyceraldehyde-3-phosphate dehydrogenase
